# Brown carbon in biomass burning aerosol enhances HONO production

**DOI:** 10.1126/sciadv.aeg9077

**Published:** 2026-11-11

**Authors:** Fengxia Bao, Lucia Iezzi, Wenjie Wang, Arianna Tronconi, Yanfang Chen, Therese S. Carter, Colette L. Heald, Kaspar Rudolf Dällenbach, David M. Bell, Markus Ammann

**Affiliations:** ^1^ PSI Center for Energy and Environmental Sciences, Paul Scherrer Institute, 5232 Villigen PSI, Switzerland.; ^2^ Institute of Atmospheric and Climate Science, ETH Zürich, 8092 Zürich, Switzerland.; ^3^ Nanjing-Helsinki Institute in Atmospheric and Earth System Sciences, Nanjing University, 215163 Suzhou, China.; ^4^ Rhodium Group, New York, NY 10019, USA.

## Abstract

Nitrous acid (HONO) is an important source of hydroxyl radicals (•OH). •OH drives ozone and aerosol formation, affecting air quality, health, and climate. Elevated HONO concentrations have been observed in biomass burning (BB) plumes. However, the mechanism remains unclear, hindering parameterization and quantification of the impacts. Here, we conducted flow tube experiments with BB aerosol samples and proxies to investigate renoxification, a process that converts nitrate into HONO and NO*_x_* (NO and NO_2_). Our results reveal that renoxification in BB aerosol is enhanced by brown carbon (BrC) through electron transfer to nitrate as an alternative to surface-enhanced nitrate photolysis. We develop parameterization to assess the potential global impacts of BrC-enhanced renoxification, which suggests that BrC enhances surface-level HONO production by up to one order of magnitude in BB-affected regions, with surface HONO production exceeding 1 part per trillion per hour in regions with high aerosol nitrate. These impacts are expected to worsen as BB intensifies in a warming climate.

## INTRODUCTION

Hydroxyl radical (•OH) is a key species in oxidizing atmospheric gases and driving aerosol formation, affecting air quality, human health, and climate (*1*–*5*). The photolysis of nitrous acid (HONO), one important component of the nitrogen cycle, is a critical source of •OH in the lower atmosphere (*6*–*8*). Emerging evidence suggests that biomass burning (BB), including prescribed burning and wildfires, leads to elevated HONO concentrations, even in areas hours to days downwind of the source (*9*–*13*). This phenomenon is increasingly concerning as BB fires are projected to increase in frequency and severity in a warming climate (*14*–*19*). A recent satellite-based analysis estimates that HONO emissions account for about two-thirds of •OH production within fresh wildfire plumes worldwide (*12*). As these plumes disperse, they carry the radical source downwind, propagating pollution far beyond fire sources (*11*). Despite its importance, HONO formation mechanisms in BB plumes remain unclear.

In early-stage aged BB plumes, direct HONO emissions may dominate or coincide with rapid secondary processes within 1 hour near fires (*20*), while secondary processes exert a more sustained influence in aged plumes hours to days downwind of the fire source, with dominant mechanisms still under debate (*11*, *21*). Inorganic nitrate, which builds up to high concentrations in aged BB plumes from the oxidation of BB NO*_x_* (NO and NO_2_) emissions (*22*), could be a potential source driving HONO formation through photochemical renoxification, which converts nitrate into HONO and NO*_x_*. In other environments (e.g., urban and marine areas), photochemical renoxification has been shown by field and laboratory measurements to be an important source of HONO and NO*_x_* (*23*–*28*). During the renoxification process, it is commonly believed that nitrate is undergoing photolysis (*23*, *26*–*30*). However, at wavelengths typical of the lower troposphere, the photolysis of nitrate in the bulk aqueous phase is known to be slow (*31*). To explain the observed renoxification, some studies suggest that it is primarily driven by enhanced photolysis of surface-bound nitrate (*23*, *26*–*30*). This enhancement may result from incomplete solvation (*32*), red-shifted and enhanced absorption (*33*–*35*), or a higher concentration of nitrate at the surface (*34*–*36*). Though, blue-shifted absorption of nitrate has also been observed in solutions with high ionic strength pertaining to aqueous aerosol particles (*37*, *38*). Some studies also argue that the surface propensity of nitrate may be overestimated (*39*) and that nitrate may be uniformly distributed or primarily located in the bulk phase (*40*, *41*). As an alternative to the enhancement of nitrate photolysis at the surface, the coexistence of organic species within the aerosol particles could contribute to renoxification. In particular, in BB aerosol, light-absorbing organic species, particularly brown carbon (BrC), are abundant and can act as photosensitizers, facilitating nitrate reduction without requiring direct nitrate photolysis. This is corroborated by recent studies that show increased renoxification of nitrate in droplets or solution in the presence of BrC proxies [e.g., vanillic acid (*42*), humic acid (*43*, *44*), 4-benzoylbenzoic acid (*45*), and sulfanilic acid (*46*)]. While the role of BrC as a photosensitizer of renoxification in aqueous-phase chemistry has been investigated, its potential to drive renoxification in BB aerosol remains unknown because of the lack of laboratory studies under relevant conditions (e.g., BB aerosol components and pH). Consequently, the parameterization and quantification of renoxification impacts in BB aerosol are still poorly constrained.

This paper aims to investigate BrC-associated photochemical renoxification using the coated wall flow tube (CWFT) technique (see Materials and Methods for details) with water-soluble organic matter (WSOM) extracted from authentic BB aerosol (generated from burning wood in a conventional wood stove) and BrC proxies [3,4-dihydroxybenzaldehyde (DHB) and benzophenone (BP)]. We mimic BB aerosol conditions by adjusting the pH, nitrate, ionic strength, BrC concentrations, and relative humidity (RH) of CWFT films while maintaining aerosol-relevant surface-to-volume ratios (see Supplementary Text, section S1). The mechanisms through which BrC influences the renoxification process are explored. In addition, we combine laboratory experiments with the output of global atmospheric model simulations to quantify the potential impact of BrC on renoxification and its contribution to global HONO production.

## RESULTS

### Role of BrC from authentic BB aerosol in photochemical renoxification

We studied photochemical processes on films of nitrate-doped samples with and without WSOM extracted from wood-burning BB aerosol using the CWFT technique (see Materials and Methods for details). Figure 1A shows that upon ultraviolet (UV) irradiation (>310 nm), samples containing WSOM and NH_4_NO_3_ ($f_{\text{WSOM}}=0.12\;g\;g^{-1}$, defined as the WSOM mass fraction relative to the total mass of WSOM and ${\text{NO}}_{3}^{-}$ in the sample; see section S2 for details) exhibited rapid formation of HONO and NO_2_, followed by a decline to a steady state. NO levels remained below the detection limit. When irradiation ceased, concentrations quickly returned to the baseline, confirming the photochemical nature of the process. We derive the total renoxification rate coefficient ($J_{{\text{NO}}_{3}^{-}\to \text{total}}$) to be 3.9 × 10^−7^ s^−1^ for the WSOM-containing nitrate samples. Here, $J_{{\text{NO}}_{3}^{-}\to \text{total}}$ is defined as the first-order renoxification rate coefficient, calculated as the total production rate of HONO and NO*_x_* divided by the nitrate concentration (see Materials and Methods for details). In contrast, control experiments with the same amount of NH_4_NO_3_ but without WSOM (Fig. 1B) produced substantially smaller amounts of HONO and NO*_x_*, yielding a lower $J_{{\text{NO}}_{3}^{-}\to \text{total}}$ of 1.2 × 10^−7^ s^−1^. This value aligns with the bulk aqueous nitrate photolysis rate coefficient of 1.4 × 10^−7^ s^−1^, calculated for our experimental irradiation conditions using nitrate absorption cross sections (*31*) and a quantum yield of 0.01, following the same approach as Shi *et al.* (*47*). The presence of WSOM ($f_{\text{WSOM}}=0.12\;g\;g^{-1}$) increases the observed $J_{{\text{NO}}_{3}^{-}\to \text{total}}$ by a factor of ∼3. This suggests that BrC in WSOM is an important chromophore contributing substantially to the photochemical reaction observed in Fig. 1A, strongly enhancing the production of HONO and NO*_x_* beyond that from direct photolysis of NH_4_NO_3_ alone.

**Fig. 1.**
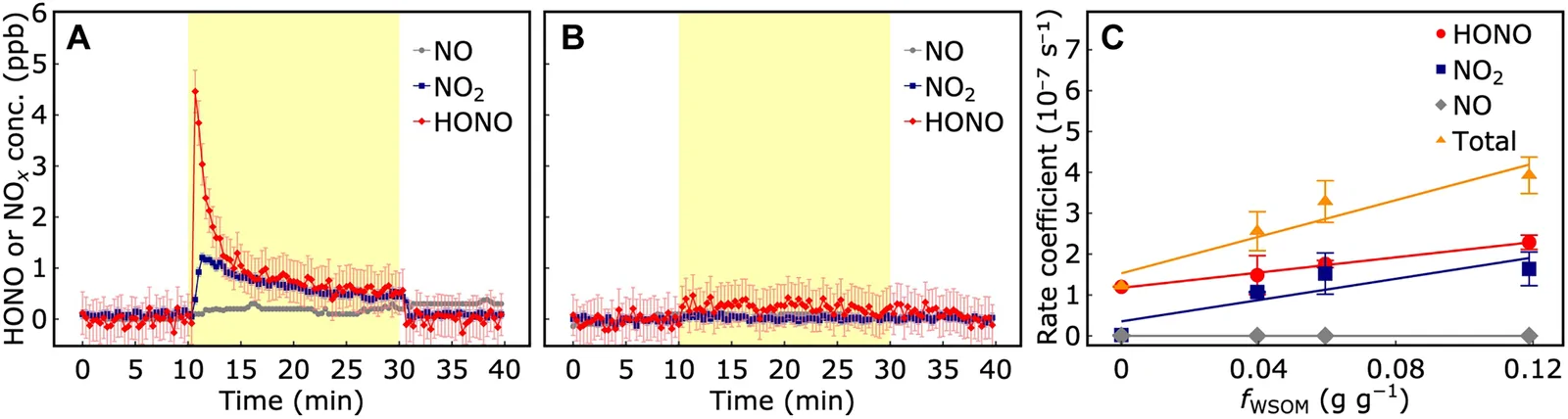
Influence of BrC on renoxification. Time profile of gas-phase HONO, NO_2_, and NO concentrations during UV irradiation (>310 nm) for (**A**) BrC sample I [BrC + NH_4_NO_3_ + (NH_4_)_2_SO_4_ + NH_4_HSO_4_] and (**B**) nitrate blank sample I [NH_4_NO_3_ + (NH_4_)_2_SO_4_ + NH_4_HSO_4_]. The yellow region denotes the period of irradiation. (**C**) First-order rate coefficients for the photochemical renoxification of nitrate, leading to the production of gas species (HONO, NO_2_, and NO) and the total first-order rate coefficient, representing their sum as a function of $f_{\text{WSOM}}$ (the WSOM mass fraction relative to the total mass of ${\text{NO}}_{3}^{-}$ and WSOM, g g^−1^) of BrC samples I to III and nitrate blank sample I. The lines represent linear fits. Error bars in (A) and (B) represent the instrument uncertainties, and error bars in (C) represent standard deviations from duplicate experiments. The experiments were conducted using an equilibrium nitrate concentration of 0.12 M at an RH of 62% and a temperature of 296 K. Details regarding the selection and atmospheric relevance of nitrate concentrations, as well as experimental control of RH and film homogeneity, are provided in section S1. Details on the initial and equilibrium composition of the samples are listed in table S1, and the $f_{\text{WSOM}}$ calculation can be found in section S2.

Hereafter, we refer to the samples with WSOM as BrC samples while noting that WSOM contains both BrC and non-BrC components. Nitrate is identified as the main nitrogen source, as the control experiment with a BrC sample without NH_4_NO_3_ yielded minimal HONO and NO*_x_* under irradiation (fig. S1). We further examined the role of BrC in photochemical renoxification by conducting CWFT experiments using a series of BrC samples with an identical amount of NH_4_NO_3_ but varying amounts of WSOM content (see Materials and Methods and table S1 for details). As shown in Fig. 1C, samples with a higher $f_{\text{WSOM}}$ exhibited increased renoxification rate coefficients. Because a higher $f_{\text{WSOM}}$ corresponds to a higher BrC content, this positive linear trend underscores the strong dependence of the renoxification process on the abundance of BrC, while non-BrC components in WSOM may also contribute through interactions with BrC, with the underlying mechanisms described later.

The experiments described above used a light source with an output of >310 nm. Given the absorption spectra of NH_4_NO_3_ and BrC (fig. S2), this irradiation can initiate both direct nitrate photolysis and BrC photosensitized reactions. BrC can therefore enhance renoxification via these two pathways: by acting as a chromophore that drives photosensitized reactions of nitrate or by potentially enhancing direct nitrate photolysis. To assess the role of BrC as a chromophore in driving renoxification, we performed additional experiments under >350-nm irradiation (fig. S3). At these wavelengths, direct nitrate photolysis is unfavorable because ${\text{NO}}_{3}^{-}$ absorbs only weakly. Potential surface-induced red shifts have been suggested; however, on the basis of reported shift magnitudes, the extension of nitrate absorption into the >350-nm range is expected to be minor (*33*–*35*). In contrast, BrC absorbs strongly and thus serves as the dominant chromophore under >350-nm irradiation. As shown in fig. S3A, nitrate photolysis in the sample without BrC (blank nitrate sample II) was not detectable, consistent with the weak ${\text{NO}}_{3}^{-}$ light absorption at these wavelengths; HONO and NO*_x_* remained below the instrumental limits of detection [LODs; 0.3 parts per billion (ppb) for HONO; 0.1 ppb for NO_2_; 0.4 ppb for NO]. In contrast, in the presence of BrC, appreciable production of HONO and NO*_x_* was observed (fig. S3B). At >350 nm, we also found a positive dependence of the rate coefficients on $f_{\text{WSOM}}$ of the BrC samples (fig. S3C). These results confirm that BrC can enhance renoxification by driving the photosensitized process, as BrC is the dominant effective chromophore under >350-nm irradiation. Combined with the results at >310 nm, these findings demonstrate that BrC photosensitization serves as a distinct enhancement pathway under atmospherically relevant irradiation, in addition to direct nitrate photolysis. Note that the rate coefficients are notably lower under irradiation at >350 nm than at >310 nm. This difference can be attributed to the reduced absorbance of BrC at higher wavelengths (fig. S2).

### Role of BrC proxy in photochemical renoxification

To further demonstrate the role of BrC in renoxification, we investigated the photochemical renoxification process using BrC proxies including DHB, a representative phenolic compound commonly found in BB aerosols (*48*, *49*), and BP, a well-established photosensitizer (*50*) included here as a mechanistic negative control (see section S3 for details). In these experiments (Fig. 2A), a higher nitrate concentration (equilibrated at 1.5 M) was used than in Fig. 1 to facilitate a more quantitative comparison. Compared to the nitrate-only samples (fig. S4), the addition of DHB leads to an increase in the total production of HONO and NO*_x_*, with HONO being substantially increased and becoming the dominant product over NO*_x_* (Fig. 2B). In contrast, the presence of BP does not lead to significant enhancement in HONO or NO*_x_* production (Fig. 2C), as confirmed by Welch’s *t* test (*P* > 0.3). Thus, not all BrC promotes renoxification, as effective BrC requires both energetically favorable excited state energy and suitable redox potentials, discussed further below. For both DHB and BP, NO exhibits a delayed rise toward a plateau compared to HONO and NO_2_. This delayed behavior may reflect secondary processes contributing to NO formation, although the underlying mechanism remains unclear. In addition, NO was detectable here in the proxy systems but not in the authentic BB BrC experiments (Fig. 1). This difference arises from the lower nitrate concentration used in Fig. 1 (equilibrated at 0.12 M), for which the expected NO concentration falls below the detection limit (0.4 ppb). Besides, the proxy systems also exhibited different temporal trends for HONO and NO_2_ (Fig. 2, B and C) compared to BrC from authentic BB aerosol (Fig. 1A). Specifically, the proxies reached their peak concentrations of HONO more slowly, followed by a gradual decline. BB BrC contains a complex mixture of BrC species, some of which may rapidly produce reactive intermediates that enhance HONO production but also decay quickly, which likely contributes to the rapid HONO peak and subsequent fast decay. Such behavior is not captured by the simple individual BrC proxy samples. Nevertheless, the selected BrC proxy DHB, which represents typical BrC in BB aerosol, effectively reproduced the enhanced effect of renoxification observed in authentic BB aerosol samples, highlighting the role of BrC in this process.

**Fig. 2.**
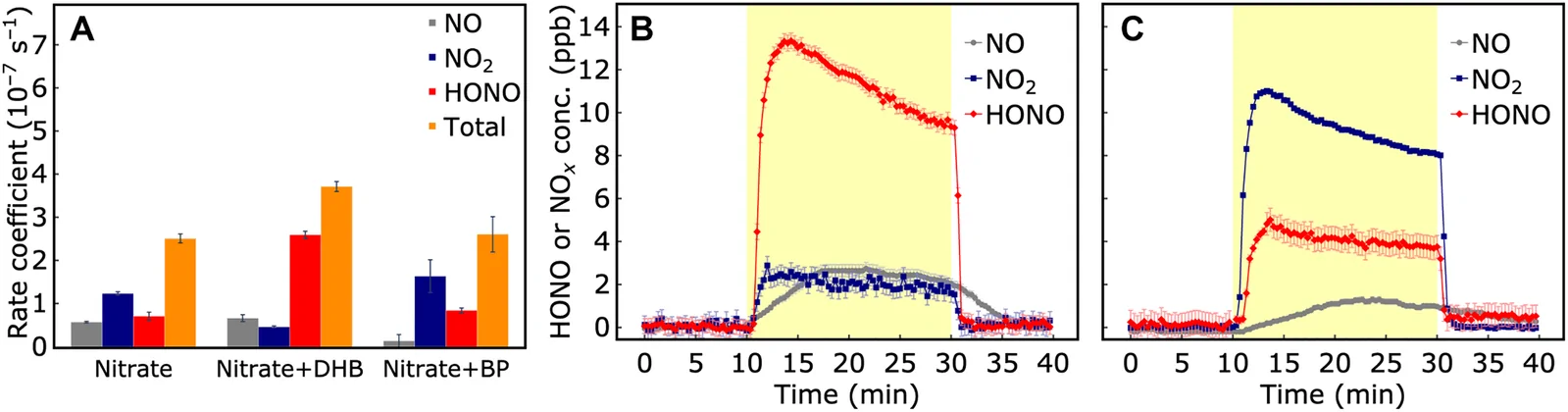
Influence of BrC proxies on renoxification. (**A**) Pseudo-steady state rate coefficients of photochemical renoxification of nitrate leading to the production of HONO, NO_2_, and NO for different samples: nitrate blank sample III, DHB sample I [DHB + NH_4_NO_3_ + (NH_4_)_2_SO_4_ + NH_4_HSO_4_], and the BP sample [BP + NH_4_NO_3_ + (NH_4_)_2_SO_4_ + NH_4_HSO_4_]. Time profile of gas-phase HONO, NO_2_, and NO concentrations during UV irradiation (>310 nm) for (**B**) DHB sample I and (**C**) the BP sample. The yellow region denotes the irradiation period in (B) and (C). Error bars in (A) represent standard deviations from duplicate experiments. Error bars in (B) and (C) represent the instrument uncertainties. The experiments were conducted using an equilibrium nitrate concentration of 1.5 M at an RH of 82% and a temperature of 291 K. More details on the initial and equilibrium composition of the samples are listed in table S1.

### Surface-enhanced nitrate photolysis versus BrC-driven renoxification

To further elucidate the roles of nitrate and BrC in the photochemical renoxification process, we conducted three sets of photochemical experiments using samples with varying compositions of nitrate and DHB: samples without DHB to understand the behavior of nitrate alone (Fig. 3, A and D); samples with DHB, maintaining a fixed DHB-to-nitrate molar ratio at 1:5 (Fig. 3, B and E); and samples with a constant concentration of DHB but a decreasing DHB-to-nitrate ratio (Fig. 3, C and F). Figure 3 (A to C) shows that the production rates increase with equilibrium nitrate concentrations ([NH_4_NO_3_]_eq_: 0.8 to 3.8 M) across different conditions, consistent with nitrate acting as the nitrogen source. Figure 3 (D and E) shows that the rate coefficients remain relatively constant across different nitrate concentrations for samples without DHB or with a fixed DHB-to-nitrate ratio. This indicates that under these conditions, nitrate concentration is not the dominant controlling factor for the renoxification rate coefficient. This is consistent with a recent chamber study on suspended nitrate salt particles by Shi *et al.* (*47*), which similarly found weak dependence of the renoxification rate coefficient on nitrate concentration. In comparison to Fig. 3 (D and E), when the DHB concentration was kept constant, smaller rate coefficients were observed at higher nitrate concentrations (Fig. 3F). Under these conditions, the DHB-to-nitrate ratio decreases, leaving fewer DHB molecules available per nitrate molecule.

**Fig. 3.**
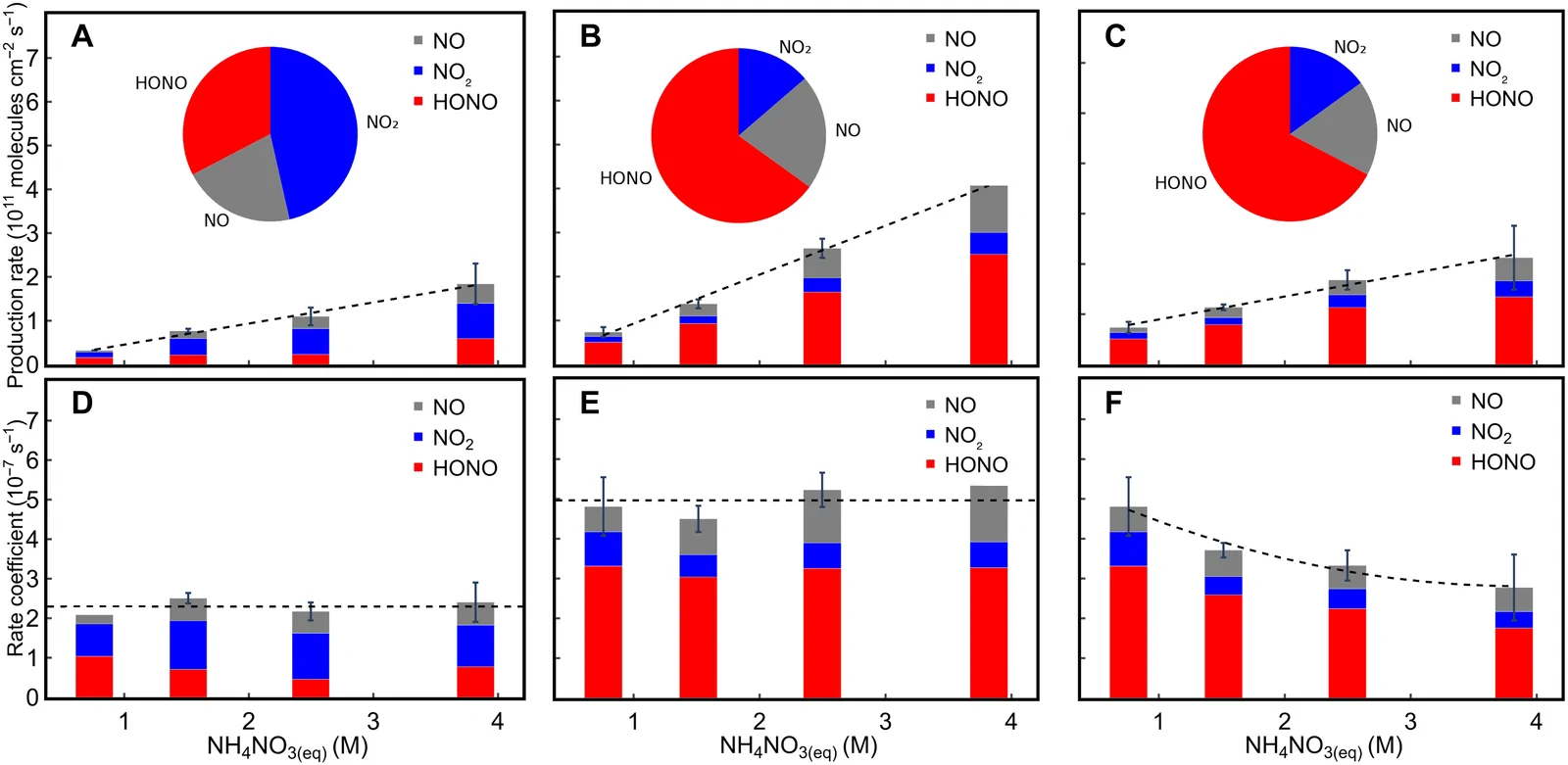
Production rates and rate coefficients of photochemical renoxification of nitrate for different samples. (**A**) Production rates and (**D**) rate coefficients at increasing nitrate concentrations ([NH_4_NO_3_]: 0.8 to 3.8 M) of nitrate blank samples III to VI without the presence of DHB. (**B**) Production rates and (**E**) rate coefficients at increasing nitrate concentrations with a fixed DHB-to-nitrate ratio of 1:5 of DHB samples II to V. (**C**) Production rates and (**F**) rate coefficients at increasing nitrate concentrations with a decreasing DHB-to-nitrate ratio (1:5 to 1:25) of DHB samples VI to IX. Pie charts in (A) to (C) illustrate the product ratio of NO, NO_2_, and HONO under each condition. Dashed lines in (A) to (F) are drawn to guide the reader’s eye. More details on the initial and equilibrium composition of the samples are listed in table S1. Error bars represent the standard deviation for measurement replicates.

We further examined the dependence of the renoxification rate coefficient on DHB concentration by combining the datasets shown in Fig. 3 (D to F) (fig. S5). At constant nitrate concentration (distinguished by color in fig. S5), higher DHB concentrations are associated with higher rate coefficients, consistent with the role of DHB as the photosensitizer. The apparent sensitivity to DHB decreases at higher nitrate concentrations, and scatter is observed when datasets across different nitrate concentrations are considered together, likely reflecting changes in the effective photosensitization efficiency of DHB under different nitrate loadings. To account for the effect of nitrate concentration, we examined the dependence of the rate coefficients on the DHB-to-nitrate ratio, which reveals a strong correlation (*R*^2^ = 0.9) across different nitrate concentrations (fig. S6), indicating that the DHB-to-nitrate ratio is the key parameter governing the rate coefficient. This ratio dependence can be explained by a kinetic analysis (see section S4 for details) of the BrC-driven photosensitization mechanism, following frameworks established for similar systems (*51*, *52*).

These results offer an alternative perspective on the inverse dependence of renoxification rate coefficients on nitrate concentration reported in previous field and laboratory studies (*23*, *26*–*29*), which has been explained by surface-catalyzed nitrate photolysis (*26*–*29*). That mechanism proposes that renoxification is driven by surface-bound nitrate. Higher rate coefficients at lower bulk nitrate concentrations are attributed to an increased fraction of nitrate residing at particle surfaces, assuming that nitrate acts as a surfactant. Our controlled experiments reproduce the inverse trend when the BrC-to-nitrate ratio decreases (Fig. 3F). Accordingly, we propose that the BrC limitation under high nitrate loading provides an alternative explanation for the observed inverse dependence. This explanation requires future field studies with simultaneous BrC and nitrate measurements, given that previous observations (*23*, *26*–*29*) were based on ambient samples where this ratio remained unconstrained. Note that the presence of DHB also affects the product distribution of renoxification (inset pie charts in Fig. 3), which will be explained in detail below.

To compare the DHB proxy system with authentic wood-burning BrC samples, we expressed DHB availability to $f_{\text{WSOM}}$, representing the DHB mass fraction relative to the total mass of DHB and ${\text{NO}}_{3}^{-}$. The dependence of the rate coefficients on $f_{\text{WSOM}}$ observed in these DHB experiments aligns with the trend previously found for the authentic BrC samples (Fig. 1C and red dots in Fig. 4). While $J_{{\text{NO}}_{3}^{-}\to \text{total}}$ for BrC from authentic BB aerosol showed a slightly more pronounced increase with increasing $f_{\text{WSOM}}$ compared to the BrC proxy DHB (Fig. 4A), the rate coefficient for HONO production ($J_{{\text{NO}}_{3}^{-}\to \text{HONO}}$) (Fig. 4B) followed substantially consistent linear trends for both BB BrC and DHB, with a strong correlation (*R*^2^ = 0.9). This confirms that DHB effectively replicates the HONO production pathway observed in BB BrC and serves as a suitable proxy. The slightly weaker dependence of $J_{{\text{NO}}_{3}^{-}\to \text{total}}$ (*R*^2^ = 0.8) on $f_{\text{WSOM}}$ may arise from additional NO*_x_* formation pathways in the more complex BB BrC, which are not fully captured by the simpler DHB system, as discussed later. Establishing this linear relationship allows us to link laboratory-derived parameterizations with global model data, thereby facilitating our subsequent global estimates. $f_{\text{WSOM}}$ was selected as the parameterization variable precisely for its compatibility with GEOS-Chem output data, enabling the direct application of these derived parameterizations. To ensure a conservative assessment, we applied the relationship between $f_{\text{WSOM}}$ and rate coefficients derived from the combined authentic BB BrC and proxy systems in our global estimate.

**Fig. 4.**
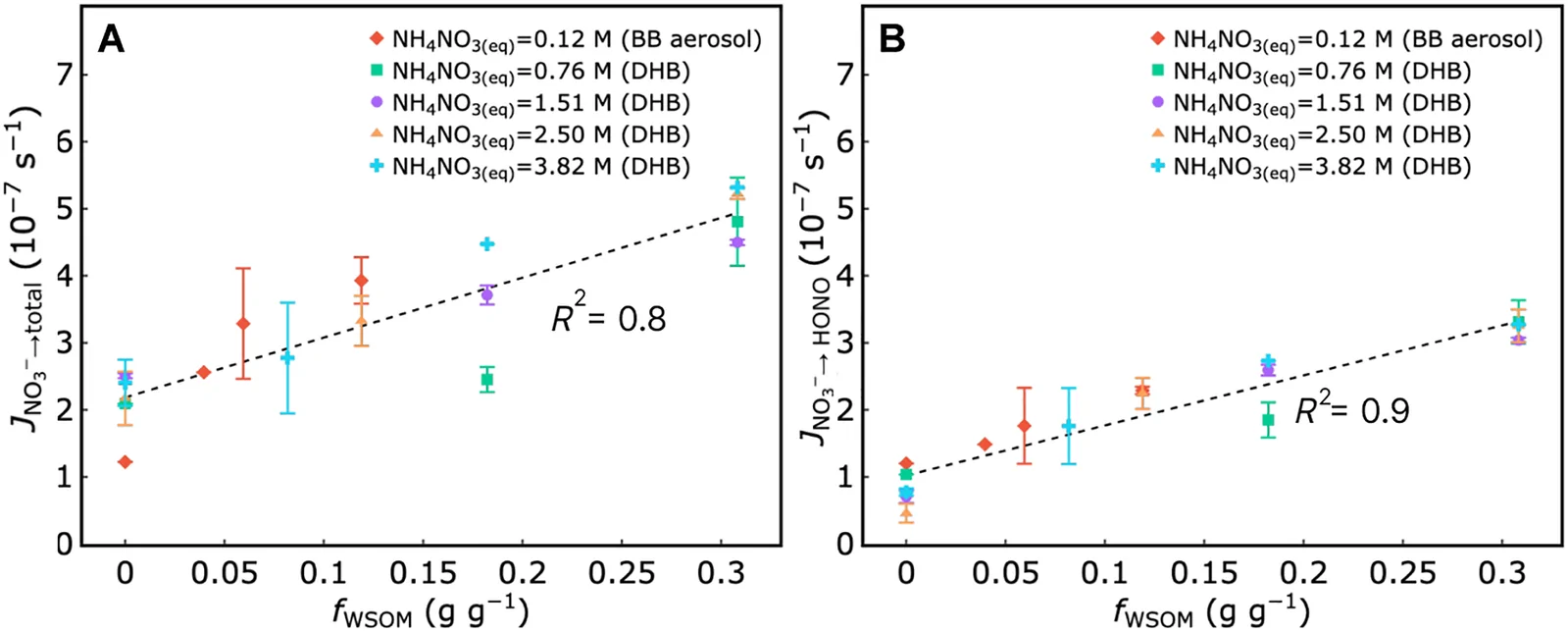
Dependence of rate coefficients for the photochemical renoxification of nitrate on the WSOM fraction $f_{\text{WSOM}}$. (**A**) $J_{{\text{NO}}_{3}^{-}\to \text{total}}$ and (**B**) $J_{{\text{NO}}_{3}^{-}\to \text{HONO}}$ are shown as functions of $f_{\text{WSOM}}$, with different symbol shapes and colors representing samples (BB BrC samples I to III, DHB samples I to IX, and nitrate blank samples I and III to VI) at different equilibrium concentrations of NH_4_NO_3_, as indicated in the legend. More details on the initial and equilibrium composition of the samples are listed in table S1. Dashed lines represent linear fits (see functions in section S6). Error bars denote standard deviations from duplicate experiments.

## DISCUSSION

### Mechanism

Our experiments confirm that renoxification can proceed without the direct photolysis of nitrate and is efficiently promoted by BrC. One unanswered question is what the specific role of BrC in renoxification is. One candidate pathway for photosensitized renoxification involves nitrate reduction by solvated electrons ($e_{\text{aq}}^{-}$) generated from BrC. Specifically, BrC is excited to the singlet state (^1^BrC*), which can undergo intersystem crossing to the triplet state (^3^BrC*) (Eq. 1). The excited BrC chromophore can undergo photoionization to generate $e_{\text{aq}}^{-}$ (Eq. 2), a process reported for organic solutes and dissolved organic matter in aquatic systems (*53*–*55*). This electron ejection has been assigned to the singlet excited state in some studies (*56*, *57*) and to the triplet in others (*58*, *59*). We therefore write Eq. 2 from a generic excited state, ${\text{BrC}}^{*}$, without ascribing it to either

BrC+hν→BrC∗1→BrC∗3 (1)

$${\text{BrC}}^{*}\to {\text{BrC}}^{\cdot +}+e_{\text{aq}}^{-}$$ (2)

Nitrate is an effective scavenger of $e_{\text{aq}}^{-}$. The reaction between nitrate and $e_{\text{aq}}^{-}$ results in the formation of ${\text{NO}}_{3}^{2-}$ (Eq. 3a), which is then converted to ${\text{NO}}_{2}$ (Eq. 4) (*60*). Subsequently, ${\text{NO}}_{2}$ undergoes reduction either by accepting $e_{\text{aq}}^{-}$ from Eq. 2 or via electron transfer from ^3^BrC*, followed by protonation to form HONO (Eq. 5 or Eq. 6) (*49*)

$${\text{NO}}_{3}^{-}+e_{\text{aq}}^{-}\to {\text{NO}}_{3}^{2-}$$ (3a)

$${\text{BrC}}^{*}+{\text{NO}}_{3}^{-}\to {\text{BrC}}^{\cdot +}+{\text{NO}}_{3}^{2-}$$ (3b)

$${\text{NO}}_{3}^{2-}+H_{2}O\to {\text{NO}}_{2}+2{\text{OH}}^{-}$$ (4)

$${\text{NO}}_{2}+e_{\text{aq}}^{-}+H^{+}\to \text{HONO}$$ (5)

NO2+BrC∗3+H+→BrC⋅++HONO (6)

Equations 1 to 6 show that BrC-driven photosensitization can increase not only the overall renoxification rate but also shift the product distribution toward HONO. This explains the increased HONO yields observed upon addition of DHB, as shown in the inset pie charts of Fig. 3, and is consistent with previous laboratory studies reporting enhanced HONO formation in the presence of BrC proxies (*42*, *49*). Notably, the HONO/NO_2_ product ratio remains relatively stable within experimental uncertainty when varying nitrate concentration at either constant DHB-to-nitrate ratio (Fig. 3E) or constant DHB concentration (Fig. 3F). This stability suggests that the secondary reduction of NO_2_ to HONO (Eqs. 5 and 6) is not solely dependent on the same photogenerated reductants (e.g., $e_{\text{aq}}^{-}$) that drive the initial nitrate reduction (Eq. 3a). Instead, it likely involves parallel or partially independent pathways, such as reduction by BrC-derived intermediates (e.g., ketyl radicals formed when excited triplet-state carbonyls abstract hydrogen from hydroxyl-containing compounds) (*49*). This diversity of reductants ensures that NO_2_ reduction remains efficient even when the demand of reductants for nitrate is high, thereby maintaining a consistent HONO-to-NO_2_ ratio across different conditions.

Equations 1 to 6 present one possible explanation for BrC-driven renoxification. Its occurrence depends on the molecular structure and redox properties of the BrC compounds. Compounds with electron-donating groups, such as phenolic (─OH) groups on aromatic rings, are more likely to generate $e_{\text{aq}}^{-}$ via photoionization (*53*, *61*). As discussed earlier, DHB and BP showed different behaviors in influencing renoxification (Fig. 2). The ─OH groups in DHB increase the electron density on the aromatic ring and lower its redox potential, enabling efficient photoionization and $e_{\text{aq}}^{-}$ ejection. In contrast, BP lacks electron-donating groups. Instead, the carbonyl group (C═O) of BP is electron-withdrawing, pulling electron density away from the aromatic ring and increasing its ionization energy. As a result, BP cannot readily eject $e_{\text{aq}}^{-}$. An alternative mechanism involving direct electron transfer from BP∗3 to ${\text{NO}}_{3}^{-}$ is not a thermodynamically feasible pathway because the reduction potential of BP∗3 [E0(BP∗3/BP⋅+)=+1.67 V versus SHE] (*62*) exceeds that of ${\text{NO}}_{3}^{-}$ $\left[E^{0}\left({\text{NO}}_{3}^{-}/{\text{NO}}_{3}^{2-}\right)=-1.10\;V\;\text{versus}\;\text{NHE}\right]$ (*46*). Another potential pathway (Eqs. 7 and 8) involving triplet energy transfer from BP∗3 to ${}^{*}{\text{NO}}_{3}^{-}$ is also not viable, given that BP’s triplet energy (288 kJ mol^−1^) is lower than that required for the excitation of ${\text{NO}}_{3}^{-}$ (*T*_1_; 332.0 kJ mol^−1^) (*46*). These considerations are consistent with our experimental observation that DHB enhances HONO production from renoxification, while BP has a negligible influence on renoxification. We note that the reduction potentials discussed here are determined under standard conditions and may not fully capture the complexity and variability of the chemical environment present in our flow tube coatings

BrC∗3+NO3−→NO3−∗+BrC (7)

$${\text{NO}}_{3}^{-*}\to {\text{NO}}_{2}+O^{-}$$ (8)

To probe whether electron transfer is involved in the reactions with DHB-like BrC, we conducted electron scavenging experiments by irradiating nitrate-doped DHB samples with and without electron scavengers in the carrier gas and, thus, in the CWFT films through Henry’s law equilibrium. We used 2-chloroethanol as an electron scavenger (Eq. 9) because it is highly water-soluble and rapidly captures electrons (*63*). The concentrations of nitrate and 2-chloroethanol in the samples were adjusted to achieve comparable first-order rates, ensuring effective competition between 2-chloroethanol and nitrate for electron scavenging within the film. Additional details are discussed in section S5. Compared with the case in the absence of 2-chloroethanol, we observed a substantial decrease in HONO formation when 2-chloroethanol was added (fig. S7, A and B), consistent with an electron-transfer step in which nitrate is reduced by an electron from the excited chromophore, either via solvated electrons (Eq. 3a) or via direct electron transfer (Eq. 3b). In addition, we tested O_2_ as an electron scavenger (Eq. 10). A slight decrease in the HONO formation rate was observed with the presence of O_2_ in the carrier gas compared to without O_2_ (fig. S7, C and D). The less pronounced influence of O_2_ is likely due to the low water solubility of O_2_, which limits its competitiveness as an electron scavenger. Note that $O_{2}^{\cdot -}$ from Eq. 10 can react with NO and NO_2_, forming nitrite (Eqs. 11 to 13), subsequently increasing HONO levels through Eq. 14. However, our observations did not show a higher HONO-to-NO_2_ ratio in the O_2_-containing atmosphere than in pure N_2_, suggesting that this mechanism is of minor importance in our DHB system. Nevertheless, we do not preclude the possibility that these pathways may play a role in BB aerosol

$${\text{ClCH}}_{2}{\text{CH}}_{2}\text{OH}+e_{\text{aq}}^{-}\to {\text{HOCH}}_{2}{\text{CH}}_{2}\text{OH}+{\text{Cl}}^{-}$$ (9)

$$O_{2}+e_{\text{aq}}^{-}\to O_{2}^{\cdot -}$$ (10)

$$O_{2}^{\cdot -}+\text{NO}\to {\text{OONO}}^{-}⇌\text{HOONO}$$ (11)

$$O_{2}^{\cdot -}+{\text{NO}}_{2}\to {\text{OONO}}_{2}^{-}\to O_{2}+{\text{NO}}_{2}^{-}$$ (12)

$$\text{HOONO}+{\text{OONO}}^{-}\to {\text{NO}}_{2}^{-}+{\text{HOONO}}_{2}\;\left(pK_{a}=6.5\right)$$ (13)

$${\text{NO}}_{2}^{-}+H^{+}⇌\text{HONO}\;\left(pK_{a}=3.3\right)$$ (14)

It is worth noting that O_2_ has also been proposed to be an energy acceptor (*62*). O_2_ can react with the triplet state of BrC via energy transfer (Eq. 15), as most BrC has higher triplet energy (typically 180 to 320 kJ mol^−1^) than required to excite O_2_ (94 kJ mol^−1^) (*62*). This returns the triplet state of BrC to its ground state, resulting in less Eq. 2 and $e_{\text{aq}}^{-}$ and thus decelerating renoxification. Meanwhile, Eq. 15 also leads to the formation of singlet oxygen (O21) (*64*), which can react with BrC components, leading to more oxidized species with weaker light absorption (*65*), thereby decreasing BrC-driven renoxification. Nevertheless, the presence of O_2_ exerts only a minor influence on renoxification under our experimental conditions

BrC∗3+O2 (Σg−3)→BrC+O21 (Δ1g) (15)

Besides BrC acting as a photosensitizer, other organic components in BB aerosol, such as electron donor–substituted phenols, may also contribute by donating electrons to BrC∗3, thereby generating ketyl radicals, which could reduce the secondary product NO_2_ to HONO or even directly reduce nitrate to NO_2_ (*49*, *66*). These ketyl radicals, as strong and short-lived reducing intermediates, may therefore influence the renoxification process. The detailed non-BrC mechanisms are beyond the scope of this study but warrant further investigation in future research. Last, our results support BrC-driven photosensitization as the dominant explanation for nitrate photoreduction in BB aerosol, rather than a mechanism attributed to enhanced nitrate concentration at the surface. Nevertheless, we cannot exclude the possibility that the surface facilitates interactions between BrC (and its photochemical intermediates) and nitrate in the underlying bulk, particularly because many BrC species (e.g., DHB) are likely surface-active. Future research systematically varying the surface-to-volume ratio is warranted to evaluate its influence on BrC-driven renoxification.

### Atmospheric implications

By combining our experiment-based parameterization with GEOS-Chem simulation results, we estimate the potential contribution of BrC to global HONO production via renoxification. Note that this global estimate is obtained by applying the experiment-based parameterization to existing GEOS-Chem output fields. It provides a first-order estimate of the potential HONO production from this pathway rather than an implementation of the mechanism in the GEOS-Chem chemical solver or a simulation of atmospheric HONO concentrations. According to our experiments, the rate coefficients of renoxification linearly increase with increasing $f_{\text{WSOM}}$ (Figs. 1C and 4). Using the linear relationships, along with the global surface-level aerosol $f_{\text{WSOM}}$ and light intensity derived from GEOS-Chem simulations for 2018, we calculate global distributions of rate coefficients of renoxification of surface-level aerosol (see details in section S6). We note that our calculation includes BrC from various combustion sources, assuming that BrC from combustion sources other than BB behaves similarly. The global annual mean surface-level $J_{{\text{NO}}_{3}^{-}\to \text{total}}^{\text{with}\_\text{BrC}}$ of aerosol is (6.02 ± 2.12) × 10^−7^ s^−1^, with its spatial distribution shown in Fig. 5A. We also calculated surface-level $J_{{\text{NO}}_{3}^{-}\to \text{total}}^{\text{no}\_\text{BrC}}$, assuming no BrC influence; that is, it reflects only direct nitrate photolysis. As shown in Fig. 3D, our experiments demonstrate that $J_{{\text{NO}}_{3}^{-}\to \text{total}}^{\text{no}\_\text{BrC}}$ values remain relatively constant at (2.29 ± 0.19) × 10^−7^ s^−1^, regardless of nitrate concentrations, in samples that do not contain BrC, comparable to previously reported values for aqueous nitrate photolysis (*27*, *29*, *67*). As mentioned earlier, much higher rate coefficients for nitrate have been reported when adsorbed on surfaces, attributed to increased absorption cross sections and red-shifted absorption spectra (*33*–*35*). Nevertheless, our conservative estimate provides a robust baseline for quantifying the enhancement effect attributable to BrC. By normalizing our experiment value of $J_{{\text{NO}}_{3}^{-}\to \text{total}}^{\text{no}\_\text{BrC}}$ with global variations in actinic flux ($J_{{\text{NO}}_{2}}$, simulated by GEOS-Chem), we generated a global map of $J_{{\text{NO}}_{3}^{-}\to \text{total}}^{\text{no}\_\text{BrC}}$ (Fig. 5B). The global average of $J_{{\text{NO}}_{3}^{-}\to \text{total}}^{\text{no}\_\text{BrC}}$ is (1.32 ± 0.47) × 10^−7^ s^−1^, which is much lower than that when the BrC influence is considered. We further define an enhancement factor ${\text{EF}}_{\text{HONO}}$ as the ratio of $J_{{\text{NO}}_{3}^{-}\to \text{HONO}}^{\text{with}\_\text{BrC}}$ to $J_{{\text{NO}}_{3}^{-}\to \text{HONO}}^{\text{no}\_\text{BrC}}$. The global mean ${\text{EF}}_{\text{HONO}}$ is 5.0 ± 2.4, underscoring the prominent role of BrC in increasing HONO formation. Figure 5C illustrates the spatial distribution of this enhancement, with ${\text{EF}}_{\text{HONO}}$ approaching 10 in tropical regions such as the Amazon Basin, Africa, and Southeast Asia, as well as in parts of Northern India and Eastern China. These areas are characterized by high temperature and frequent BB activity, contributing to elevated BrC. The much higher EFs for ambient aerosols documented elsewhere (*23*, *26*, *27*) would lead to even higher HONO formation rates. Here, we quantify the atmospheric implications of the experimentally constrained BrC-driven renoxification pathway characterized in this study.

**Fig. 5.**
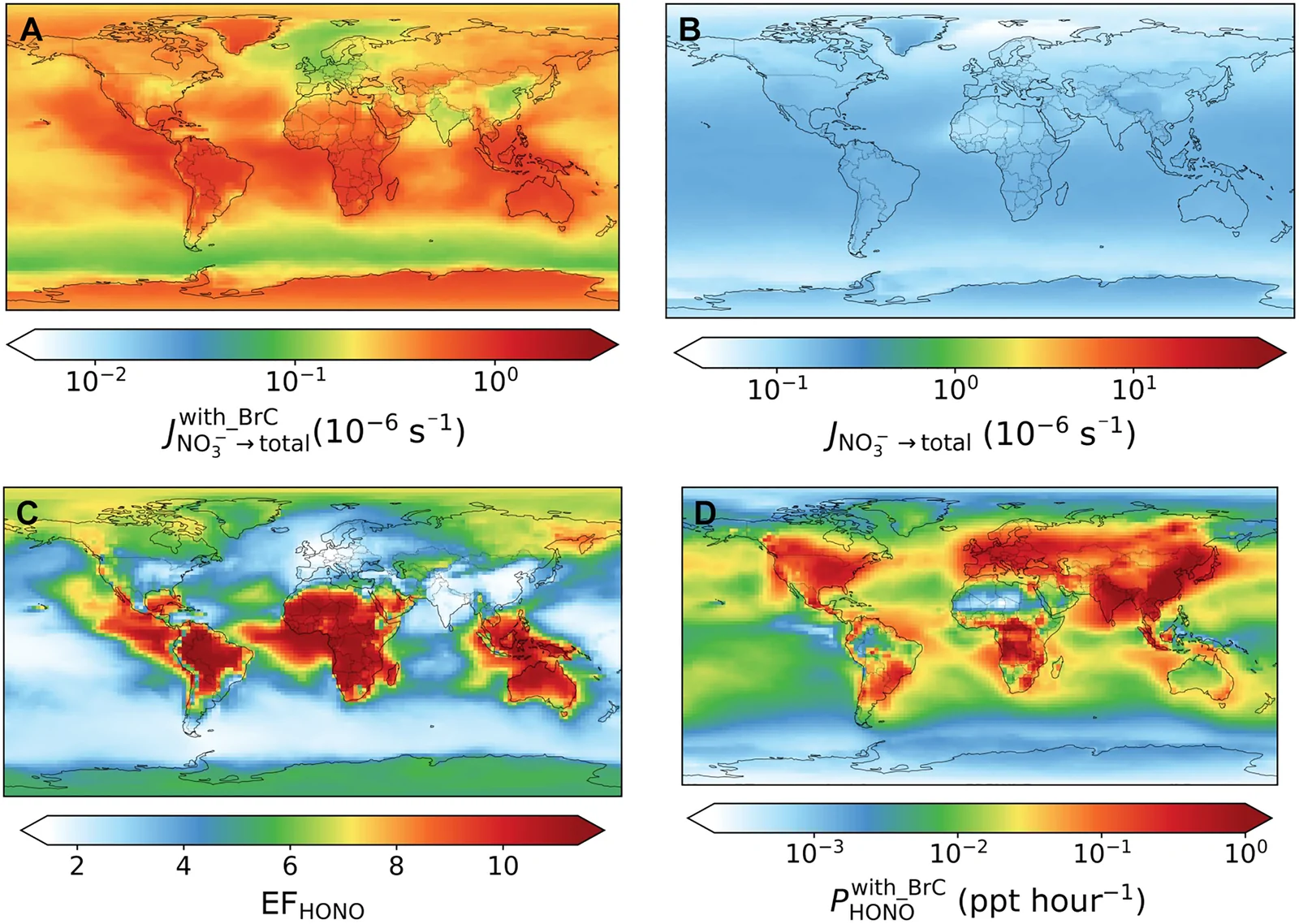
Global distributions of annual mean surface-level renoxification rate coefficients, ${\text{EF}}_{\text{HONO}}$, and HONO production rate based on our experiment-based parameterization combined with GEOS-Chem simulation outputs for 2018. Distribution of the annual mean surface-level $J_{{\text{NO}}_{3}^{-}\to \text{total}}$ (**A**) with BrC in aerosols and (**B**) without BrC in aerosols. (**C**) Distribution of annual mean surface-level ${\text{EF}}_{\text{HONO}}$. (**D**) Distribution of annual mean surface-level HONO production rate, $P_{\text{HONO}}$, with BrC in aerosols. These values are derived by multiplying the monthly mean surface-level $J_{{\text{NO}}_{3}^{-}\to \text{total}}$ with the monthly mean surface-level aerosol nitrate concentrations and then averaging the resulting values annually.

We further estimated the HONO production rate by multiplying the surface $J_{{\text{NO}}_{3}^{-}\to \text{HONO}}^{\text{with}\_\text{BrC}}$ with simulated aerosol nitrate concentrations (fig. S8). Figure 5D shows that $P_{{\text{NO}}_{3}^{-}\to \text{HONO}}^{\text{with}\_\text{BrC}}$ exceeds 1 part per trillion (ppt) hour^−1^ in regions with both high aerosol nitrate concentrations (fig. S8) and strong BrC emissions (fig. S9), such as Central Africa, Northern India, Eastern China, and parts of Southeast Asia. This is a global annual mean; field studies report much higher HONO production under local, short-lived conditions, from tens to hundreds of ppt hour^−1^ in daytime surface air (*25*) to order 10^4^ ppt hour^−1^ in wildfire plumes (*11*, *20*), so a global annual mean falls well below these local rates. To compare directly at the plume scale, we evaluated the parameterization against these wildfire plume observations of Peng *et al.* (*11*). Applying the parameterization to their measured aerosol nitrate and water-soluble organic fraction of each plume transect (see details in section S7), we find that including BrC increases the predicted HONO production by a factor of 8.2 ± 0.1 relative to particulate nitrate photolysis alone. Although the predicted production remains below the inferred secondary HONO source, the discrepancy decreases from approximately a factor of 700 to a factor of 86 (fig. S10). The predicted production tracks the inferred secondary HONO source more closely for aged plumes (physical age >3 hours; Spearman ρ=0.81, n=87) than for fresh plumes (<3 hours; ρ=0.47, n=95), consistent with the proposed pathway acting as a secondary HONO source that develops with photochemical aging. The comparison indicates that the laboratory-derived parameterization systematically underestimates the inferred secondary HONO source under the WE-CAN wildfire conditions. Both sides of this comparison carry uncertainty: The inferred secondary HONO source is a residual of the gas-phase HONO budget and depends on gas-phase rate coefficients that have recently been revised (*68*, *69*), while our parameterization reflects only the water-soluble BrC fraction and a deliberately low organic content for film homogeneity, with differences in phase, mixing, humidity, and temperature adding further uncertainty. The global estimate presented here is therefore best interpreted as a conservative first-order estimate of this pathway.

Our findings indicate that BB aerosol is a potentially important, previously underestimated source of HONO. By acting as a donor of $e_{\text{aq}}^{-}$, BrC∗3 enables BB aerosol to efficiently reduce nitrate into HONO while being oxidized itself. The subsequent photolysis of HONO produces •OH, which can enhance the oxidative capacity and ozone production of the plume as it ages downwind. Meanwhile, the oxidized BrC may undergo further aging processes, influencing aerosol aging and radiative forcing, which warrants future investigation. This mechanism has the potential to help explain the persistent HONO production observed in aged plumes (*11*) and reframes BB plumes as active photochemical reactors that couple reactive nitrogen recycling with aerosol aging. Apart from the immediate harmful impacts of fires, this work highlights their long-term effects on air quality and climate through the long-range transport of emissions and reactive species. Guided by this framework, we highlight that incorporating BrC-enhanced renoxification of BB aerosol into future global atmospheric models will be crucial for accurately predicting atmospheric chemistry, evaluating air quality and developing effective climate policies. This is particularly urgent as climate warming increases the frequency and intensity of fires (*14*–*19*), amplifying BrC-enhanced renoxification and aggravating its impacts.

## MATERIALS AND METHODS

### Materials

The following chemicals were used: ammonium nitrate (≥99.5%, Sigma-Aldrich), ammonium sulfate (≥99.5%, Sigma-Aldrich), ammonium hydrogensulfate (≥98.5%, ROTH), BP (≥99%, Sigma-Aldrich), 2-chloroethanol (≥99%, Sigma-Aldrich), and DHB (≥98%, Sigma-Aldrich). Ultrapure water [MQ H_2_O, Arium PRO Sartorius, <5-ppb total organic carbon (TOC), 0.055 μS cm^−1^] was taken fresh to prepare the coating solution and to fill the water bubblers used as the humidifier. NO was delivered from a gas cylinder [Messer, 10 parts per million (ppm)].

### CWFT

A series of experiments were conducted in a photochemical flow system with CWFT. The CWFT, made of DURAN glass, has an inner diameter of 1.2 cm and a length of 50 cm. A coating solution (1 ml) was injected into the sandblasted tube, which was then rotated and gently shaken to ensure full and even coverage of the inner wall. An excess solution was allowed to drain into a volumetric flask, and the initial coating volume was estimated to be ∼0.3 ml on the basis of the collected volume. The tube was then inserted into a double-jacketed glass cell connected to a circulating water bath to maintain a constant temperature. Before each experiment, the tube was cleaned using a sequential procedure (acetone, ethanol, NaOH, HCl, deionized water, and Milli-Q water) to minimize surface impurities and ensure reproducible conditions. A carrier gas flow containing N_2_ and O_2_ passed through the CWFT, allowing the water activity of the coating to equilibrate at a specific RH (see table S1 for specific conditions). Under equilibrium conditions, the water content decreases substantially. Using the E-AIM model, we estimate an equilibrium film volume of 24 to 40 μl (depending on RH and composition). Distributed over a tube surface area of 188.5 cm^2^, this corresponds to a mean film thickness of δ≈1.3 to 2.1 μm, yielding a surface-to-volume ratio of $S/V\approx \left(4.8\;\text{to}\;7.7\right)\times 10^{5}$ m^−1^, equivalent to spherical particles with diameters of ∼8 to 13 μm, characteristic of coarse-mode aerosol particles. Besides, at these CWFT thicknesses (δ≈1.3 to 2.1 μm), aqueous-phase diffusion across the film occurs on millisecond timescales, much faster than the gas residence time in the CWFT. The CWFT therefore probes heterogeneous aerosol photochemistry in a well-mixed, RH-equilibrated thin film, minimizing the transport limitations characteristic of bulk solutions while maintaining an aerosol-relevant surface-to-volume ratio.

In addition to the aerosol-relevant surface-to-volume ratio and efficient diffusion in the CWFT film, the CWFT configuration offers the critical advantage of providing sufficient reactant mass to generate detectable product signals while maintaining an optically thin layer. Nevertheless, the setup is not designed to isolate surface or curvature effects; consequently, the influence of these factors and the extension of our results to fine-particle regimes remain open questions and warrant further study. Our focus is on liquid-phase aerosol photochemistry, for which the CWFT technique is well suited and has been shown to probe bulk photochemical processes under optically thin conditions, yielding results consistent with aerosol flow tube measurements when differences in diffusion length are accounted for (*50*).

### Sampling of BB aerosol and extraction of WSOM

To generate BB aerosol, beech wood (sourced from a local forestry company in Würenlingen, Switzerland) was burned in a stove facility that is part of the Atmospheric Simulation Chambers and Reactors at the Paul Scherrer Institute (*70*). The wood was ignited with fire starters, and the burning emissions were pulled into a chimney. After the starters burnt away, the emissions were diluted via an injection diluter (Dekati, 10:1 dilution), using stainless steel lines (heated to 150°C), into a custom-made filter-based particulate sampler. The BB aerosol samples were collected during the smoldering phase at a flow rate of 10 liters min^−1^ for 20 min using quartz fiber filters (47 mm, Pallflex), which had been prebaked for 6 hours at 500°C. Immediately after collection, the filters were stored at −20°C in the dark to prevent chemical aging. To minimize solution-phase aging effects, fresh extraction was performed on the day of each experiment. A defined portion of the filter was extracted with ultrapure water in an amber vial by shaking for 15 min. The WSOM extracts were then filtered through a 30-mm-diameter polytetrafluoroethylene filter with a 0.2-μm pore size using a syringe. Variations in WSOM concentration for the kinetic experiments were achieved by diluting this fresh extract with ultrapure water. Note that BB emissions can vary substantially depending on combustion conditions, oxygen availability, wood moisture content, and wood species (*71*, *72*). The conclusions drawn from our study are robust within the context of smoldering-phase emissions from beech wood combustion. Beech is recognized as a representative genus of forests, commonly found throughout temperate regions worldwide. Nevertheless, caution is warranted when generalizing our findings to different biomass types and other combustion conditions (*73*, *74*).

### Quantification and light absorption measurements

The water-soluble organic carbon (WSOC) of WSOM extracts of BB filter samples was measured by a TOC analyzer (TOC-LCPH, Shimadzu). Details on the conversion of WSOC to WSOM, as well as the calculation of the WSOM fraction in aerosols ($f_{\text{WSOM}}$), are provided in section S2.

The light absorption of the BB aerosol water extract, as well as that of aqueous solutions of ammonium nitrate and DHB and a 1:1 (water:acetone) solution of BP, was measured using a UV-visible spectrometer (AvaSpec-ULS2048XL-EVO) at their native pH without additives. Because the extraction protocol described above ensures the compositional consistency of the WSOM obtained from the biomass-burning filter set, the spectra of the BB aerosol water extracts were considered representative of the BB BrC samples used in the CWFT experiments. Note that measurable but limited red shifts between pH 4 (CWFT) and pH 6 (spectral measurement) in the optical properties of BrC from water extracts of biomass-burning aerosol have been reported (*75*). The spectra exhibit similar trends, with broad absorption across the wavelengths of our lamps. Overall, our measurements are qualitative, focusing on the identification of absorption features rather than precise quantification of absorbance.

### Gas phase monitoring

Gaseous products NO*_x_* and HONO released during the experiment were flushed out of the photochemical flow system by the carrier gas and were detected online. A chemiluminescence NO*_x_* analyzer (CLD Teledyne T200) was used to detect NO. Two separate ICAD [iterative cavity-enhanced differential optical absorption spectroscopy (CE-DOAS), Airyx GmbH] measurement systems (*76*) were used to detect NO_2_ and HONO. CE-DOAS has been widely used to measure in situ atmospheric trace gases over the past decades. The ICAD is a spectroscopic instrument that enhances CE-DOAS using an iterative algorithm. It can directly measure in situ concentrations of HONO and NO_2_.

The LOD for all gas-phase instruments was determined as three times the standard deviation of the signal measured in zero air (3σ). The LODs were 0.1 ppb for NO_2_, 0.3 ppb for HONO, and 0.4 ppb for NO all at a time resolution of 20 s. Regarding calibration, the NO*_x_* analyzer was calibrated using standard NO gas cylinders (Messer). The ICAD instruments rely on an absolute measurement principle using specific molecular absorption cross sections and effective path lengths determined by mirror reflectivity; therefore, they do not require daily calibration with span gases. The zero point for all instruments was determined by measuring ultrapure synthetic air (N_2_ and O_2_).

### Calculation of renoxification rate coefficient

The pseudo-steady state ($P_{\text{gas}\_\text{species}}$) production rates (mol s^−1^) of photochemical renoxification of nitrate leading to the gas species (HONO, NO_2_, and NO) were calculated using the following equations

$$P_{\text{gas}\_\text{species}}=\frac{10^{-9}\cdot F_{g}\cdot c_{\text{gas}\_\text{species}}}{V_{m}}$$ (16)

$$P_{\text{total}}=P_{\text{HONO}}+P_{{\text{NO}}_{2}}+P_{\text{NO}}$$ (17)

$c_{\text{gas}\_\text{species}}$ (ppb) represents the average concentration of gas species during the last 3 min of the irradiation period when a pseudo-steady state is observed, $F_{g}$ is the flow rate of the carrier gas (liters s^−1^), and $V_{m}$ is the molar volume of the gas at 1 atm (liters mol^−1^).

The pseudo-steady first-order rate coefficient ($J_{{\text{NO}}_{3}^{-}\to \text{gas}\_\text{species}}$, s^−1^) for the photochemical renoxification of nitrate, leading to the production of gas species (HONO, NO_2_, and NO), and the total rate coefficient ($J_{{\text{NO}}_{3}^{-}\to \text{total}}$, s^−1^), representing their sum, were calculated using the following equations

$$J_{{\text{NO}}_{3}^{-}\to \text{gas}\_\text{species}}=\frac{P_{\text{gas}\_\text{species}}}{N_{{\text{NO}}_{3}^{-}}}$$ (18)

$$J_{{\text{NO}}_{3}^{-}\to \text{total}}=J_{{\text{NO}}_{3}^{-}\to \text{HONO}}+J_{{\text{NO}}_{3}^{-}\to {\text{NO}}_{2}}+J_{{\text{NO}}_{3}^{-}\to \text{NO}}$$ (19)

where $N_{{\text{NO}}_{3}^{-}}$ (mol) is the amount of ${\text{NO}}_{3}^{-}$ in the CWFT films.

### GEOS-Chem simulations

GEOS-Chem (http://geos-chem.org/) model simulations from a previous study (*77*) were used to derive global surface $f_{\text{WSOM}}$, aerosol nitrate concentration ($C_{{\text{NO}}_{3}^{-}}$), and light intensity ($J_{{\text{NO}}_{2}}$) to estimate renoxification rates. These simulations were performed for 2018 using version 12.3.0 of the model and driven by assimilated meteorology from MERRA-2 at a horizontal resolution of 2° by 2.5°. Further details on these simulations and the model configuration can be found in a previous study (*77*). See section S6 for details on calculating $f_{\text{WSOM}}$ and renoxification rates on the basis of the model simulations.

## Supplementary Materials

### This PDF file includes:

Supplementary Text Figs. S1 to S10 Table S1 References
