# Supplementary material for: Brown carbon in biomass burning aerosol enhances HONO production
Source: Sci Adv. 2026 Nov 11;12(46):eaeg9077. doi: 10.1126/sciadv.aeg9077 (PMC13754036; doi:10.1126/sciadv.aeg9077)
Supplement: Supplementary file 1 [file sciadv.aeg9077_sm.pdf]

Science Advances
AAAS

Supplementary Materials for

Brown carbon in biomass burning aerosol enhances HONO production

Fengxia Bao *et al.*

Corresponding author: Fengxia Bao, fengxia.bao@psi.ch

Sci. Adv. **12**, eaeg9077 (2026)
DOI: 10.1126/sciadv.aeg9077

This PDF file includes:

Supplementary Text
Figs. S1 to S10
Table S1
References

Supplementary Text

1. Nitrate molarity, relative humidity (RH), and sample homogeneity in coated wall flow tube (CWFT) experiments

The range of equilibrium nitrate molarities (0.1-3.8 M) used in this study was selected to ensure that the renoxification rate coefficients could be reliably measured while also maintaining relevance to biomass burning (BB) aerosol. Elevated nitrate levels have been observed in BB plumes (*22, 78–80*). For instance, Song *et al.* (*79*) reported NO_3^- concentrations as high as (42 ± 8) μmol m^{-3} (2610 ± 502) μg m^{-3} in prescribed fire plumes. Meanwhile, Peng *et al.* (*22*) observed that ∼ 29% of emitted NO_x was converted into nitrate, up to 20 ppb (50.8 μg/m^3) within 1 hour of plume aging from wildfires. To compare the nitrate levels of BB aerosol to the molarities used in our study, we estimated the aerosol nitrate molarity indirectly based on measurements of wildfires. To this end, we use the $PM_{2.5}$ concentration of (32 ± 21) μg m^{-3} from 8 wildfire studies ((*80*) and references therein) and assume an aerosol density of 1.4 g cm^{-3}, a median value within the reported range of 0.85-1.92 g cm^{-3} (*81–83*), which enables the estimation of the aerosol aqueous phase volume as (0.023 ± 0.015) μL m^{-3}. Further, adopting the average nitrate concentrations (14 ± 4) μg m^{-3} from four near-field wildfires (*22*) yields the nitrate molarity (9.9 ± 9.3) M. This value includes the range used in our experiments, confirming the atmospheric relevance of the nitrate concentrations selected. While lower nitrate concentrations may occur in very clean environments, concentrations below 0.1 M were not examined here due to measurement limitations. Our results are intended to represent conditions characteristic of BB aerosol.

To maintain the homogeneity of aqueous films and prevent crystalline precipitation, all experiments were conducted under carefully controlled RH conditions. Before each experiment, the coatings were equilibrated at the target RH to avoid crystallization, which was confirmed by visual inspection. For the mechanistic investigation using DHB (Fig. 3), we intentionally used a higher RH (∼82%) to maximize dissolution and promote homogeneity. For experiments using BB WSOM water extracts (Fig. 1), we used an RH of ∼62% to reflect the global average conditions relevant to our parameterization for global estimates. Also note that aerosol WSOM mixed-component systems tend to have lower efflorescence RH than single-component systems (*84*), which prevents

crystallization. Besides, the renoxification rate coefficient exhibited a consistent, linear increase with the water-soluble organic matter (WSOM) fraction (f_{WSOM}) of samples over the range of concentrations examined (Fig. 4), suggesting that the reaction kinetics are independent of concentration within this range. Nevertheless, complete homogeneity of the organic distribution cannot be guaranteed. Although our method ensures dissolution and avoids macroscopic phase separation, we cannot completely rule out the possibility of surface enrichment by amphiphilic BrC components.

2. Calculation of f_{WSOM} for CWFT samples

f_{WSOM} (g g^{-1}) was calculated by Eq. S1 as the mass fraction of WSOM (m_{WSOM}) to the sum of the total mass of WSOM (m_{WSOM}) and NO_3^- ($m_{\mathrm{NO_3^-}}$).

$$f_{\mathrm{WSOM}} = \frac{m_{\mathrm{WSOM}}}{m_{\mathrm{NO_3^-}} + m_{\mathrm{WSOM}}} \tag{S1}$$

m_{WSOM} of BrC samples (f_{WSOM}) was estimated based on the total of organic carbon (TOC) measurement. To convert the measured water-soluble organic carbon (WSOC) to total WSOM, a $ratio_{OM:OC}$ representing the mass ratio of organic matter to organic carbon of 1.64 was applied, based on previous studies of BB aerosol (*85*). The total WSOM mass (m_{WSOM}) was then calculated as:

$$m_{WSOM} = C_{WSOC}^{m} \times V \times ratio_{OM:OC} \tag{S2}$$

where C_{WSOC}^{m} (g mL^{-1}) is the measured initial mass concentration of water-soluble TOC in the coating solution, and V (mL) is the initial volume used to coat the CWFT.

Additionally, the molar concentration of WSOM (C_{WSOM}^{M}, mol L^{-1}) was estimated in table S1 as:

$$C_{WSOM}^{M} = \frac{C_{OC}^{m} \times ratio_{OM:OC}}{200} \tag{S3}$$

where 200 is the typical molecular weight for BrC compounds (*86*).

3. Rationale for choice of BrC proxies

In addition to authentic WSOM extracted from BB aerosol, we used 3,4-dihydroxybenzaldehyde (DHB) and benzophenone (BP) as BrC model compounds. DHB was chosen as a representative phenolic carbonyl, a class of chromophores identified in molecular-level analyses of BB organic aerosol (*48*) that is expected to efficiently promote renoxification. In contrast, BP was used as a mechanistic negative control. It is a well-known aromatic ketone photosensitizer (*50*); its redox potential and triplet energy make participation in the photosensitized renoxification pathway thermodynamically unlikely under our experimental conditions. The reaction mechanisms are discussed in detail in the Mechanism section of the main text. These mechanistic characteristics, combined with their suitable solubility and stability in our coating solutions, make DHB and BP practical and representative proxies for probing BrC-driven renoxification in the CWFT experiments.

4. Kinetic Analysis of Photosensitized Renoxification

The observed dependence of renoxification rate coefficients on the DHB-to-nitrate ratio can be explained by the kinetics of BrC-driven photosensitization. Photoexcitation of DHB generates excited states at a rate:

$$R_{\mathrm{form}} = J_{\mathrm{abs}}[\mathrm{DHB}] \tag{S4}$$

where J_{abs} represents the effective absorbed-photon rate per DHB molecule, which depends on the actinic flux of the lamp, the absorption cross-section of DHB, and the quantum yield of the excited state. Once formed, DHB^* can be deactivated through two competing pathways: productive quenching by nitrate leading to renoxification (rate coefficient k_q), and unproductive processes including intrinsic decay and quenching by other species (effective rate coefficient k_0). This competitive framework follows a similar kinetic competition as reported for other photosensitized processes involving excited organic chromophores (*51, 52*). The total loss rate is:

$$R_{\mathrm{loss}} = (k_0 + k_q[\mathrm{NO_3^-}])[\mathrm{DHB}^*] \tag{S5}$$

Under continuous irradiation, DHB^* reaches a steady state where formation equals loss. Applying the steady-state approximation, $\frac{d[\mathrm{DHB}^*]}{dt} \approx 0$, we obtain:

$$[\mathrm{DHB}^*] = \frac{J_{\mathrm{abs}}[\mathrm{DHB}]}{k_0 + k_q[\mathrm{NO}_3^-]} \tag{S6}$$

The renoxification products (HONO and NO_x) are generated through the productive quenching pathway at a rate:

$$R_{\mathrm{prod}} = k_q[\mathrm{NO}_3^-][\mathrm{DHB}^*] = \frac{J_{\mathrm{abs}}[\mathrm{DHB}]k_q[\mathrm{NO}_3^-]}{k_0 + k_q[\mathrm{NO}_3^-]} \tag{S7}$$

The experimentally measured rate coefficient J_{obs} is defined as the production rate normalized by the nitrate concentration:

$$J_{\mathrm{obs}} = \frac{R_{\mathrm{prod}}}{[\mathrm{NO}_3^-]} = \frac{J_{\mathrm{abs}}[\mathrm{DHB}]k_q}{k_0 + k_q[\mathrm{NO}_3^-]} \tag{S8}$$

This expression directly predicts the inverse dependence of J_{obs} on nitrate concentration observed in Fig. 3F when DHB is held constant. It also provides an alternative mechanistic explanation for previous field and laboratory studies (*23, 26–29*) where higher ambient nitrate concentrations correlated with lower renoxification rate coefficients.

When nitrate-mediated quenching dominates ($k_q[\mathrm{NO}_3^-] \gg k_0$):

$$J_{\mathrm{obs}} \approx J_{\mathrm{abs}}\frac{[\mathrm{DHB}]}{[\mathrm{NO}_3^-]} \tag{S9}$$

the rate coefficient scales directly with the DHB-to-nitrate ratio, consistent with the linear dependence of rate coefficients on this ratio observed in fig. S6.

In contrast, when unproductive decay dominates ($k_0 \gg k_q[\mathrm{NO}_3^-]$):

$$J_{\mathrm{obs}} \approx J_{\mathrm{abs}}\frac{k_q}{k_0}[\mathrm{DHB}] \tag{S10}$$

the rate coefficient depends primarily on photosensitizer concentration. While this regime may occur in clean environments with very low nitrate levels, the nitrate concentrations relevant to our experiments (0.12–3.82 M) and BB aerosol (as discussed in section S1) satisfy the condition that $k_q[\mathrm{NO}_3^-] \gg k_0$. Thus, Eq. S9 represents the dominant kinetic regime under our conditions.

5. Electron scavenger experiments

To investigate the role of e_{aq}^- in renoxification, we conducted scavenger experiments using 2-chloroethanol. A dedicated N_2 line was either routed through the headspace of a vessel containing

pure 2-chloroethanol or routed to a bypass line (no 2-chloroethanol), allowing rapid switching between "with scavenger" and "without scavenger" conditions. This 2-chloroethanol-containing (or bypass) N_2 stream was then mixed upstream with the humidified N_2 gas before entering the CWFT; because the two streams were mixed at a 1:1 flow ratio, the 2-chloroethanol vapor was diluted two-fold upon entering the flow tube. To ensure thermal stability and a constant 2-chloroethanol source strength, the 2-chloroethanol reservoir was maintained in an ice bath. At this temperature, the saturation vapor pressure is 1.8 mbar; following dilution, the partial pressure entering the flow tube was 0.9 mbar ($P_{\text{2-chloroethanol}}$).

Based on the Henry's law solubility constant (H^{cp}) of 1.3 M mbar^{-1} at 298 K (*87*) (assumed applicable to the 296 K CWFT temperature), we estimate an equilibrium aqueous 2-chloroethanol concentration of ~1.2 M in the CWFT film ($C_{eq} = H^{cp} \times P_{\text{2-chloroethanol}}$). In contrast, the equilibrium concentration of NO_3^- in the CWFT film was 0.1 M. Although the rate coefficient for the reaction of 2-chloroethanol with e_{aq}^- (2×10^8 M^{-1} s^{-1}) is substantially lower than that for NO_3^- (9.7×10^9 M^{-1} s^{-1}) (*63*), the molar excess of the scavenger compensates for this difference. The resulting pseudo-first-order scavenging rate for 2-chloroethanol (2.4×10^8 s^{-1}) is of comparable magnitude to that for NO_3^- (9.7×10^8 s^{-1}); therefore, 2-chloroethanol can compete effectively with NO_3^- for e_{aq}^-.

6. Estimate the contribution of BrC to HONO and NO_x production via renoxification based on the model simulations

To evaluate the global contribution of BrC to HONO and NO_x production via renoxification, we integrated our laboratory findings with the GEOS-Chem model (v.12.3.0) simulations. Details on primary organic aerosol simulation are described and tested in a previous study (*77*). The experimentally derived relationship between the renoxification rate coefficient and f_{WSOM} in aerosols was parameterized as linear functions (Fig. 4) with normalization of light intensity:

$$J_{NO_3^- \to HONO} = (7.46 \times 10^{-7} \times f_{WSOM} + 1.02 \times 10^{-7}) \times \frac{J_{NO_2}}{J_{NO_2}^{exp}} \quad (R^2 = 0.9) \qquad \text{(S11)}$$

$$J_{NO_3^- \to total} = (8.93 \times 10^{-7} \times f_{WSOM} + 2.18 \times 10^{-7}) \times \frac{J_{NO_2}}{J_{NO_2}^{exp}} \quad (R^2 = 0.8) \qquad \text{(S12)}$$

where f_{WSOM} is derived from GEOS-Chem simulations (details shown below). Monthly mean values are used for calculation here. J_{NO_2} is the photolysis rate of NO_2 simulated using GEOS-

Chem, representing atmospheric light intensity, $J^{exp}_{\mathrm{NO_2}}$ is ~0.01 s^{-1}, representing the photolysis rate of NO_2 of laboratory light condition of lamp 1 (> 310 nm), which is calculated using the measured actinic flux of the light source used (fig. S2), the absorption cross-section of NO_2 (*88*) and the quantum yield (*89*) integrated over the relevant wavelength range. $\frac{J_{\mathrm{NO_2}}}{J^{exp}_{\mathrm{NO_2}}}$ is the correction factor that scales the experimental renoxification rate coefficients to reflect atmospheric light conditions. $J_{\mathrm{NO_2}}$ is computed online by Fast-JX (*90–92*), which calculates photolysis rates from the simulated aerosol fields and their wavelength-dependent optical properties, so it already encodes the local actinic flux and its attenuation by aerosol and plume opacity at the model resolution. Grid-scale light attenuation, including the darkening of absorbing plumes, is therefore carried into the parameterization through this factor, on the same basis as $J_{\mathrm{NO_2}}$ is used for gas-phase NO_2 photolysis. Nevertheless, the grid-mean field does not resolve the light gradient within an individual optically thick plume (sub-grid, particle-level heterogeneity), which remains a source of uncertainty.

We note that this parameterization is derived from controlled laboratory experiments designed to mimic BB aerosol conditions (see Materials and Methods as well as section S1). Environmental factors such as temperature, aerosol phase state, and relative humidity may introduce heterogeneity in the rate coefficient under ambient conditions, warranting future investigation under a broader range of conditions and field validation.

6.1 Calculation of f_{WSOM} based on the model simulations f_{WSOM} (g g^{-1}) was calculated by Eq. S13 as the fraction of the mass concentration of WSOM (c_{WSOM}) to the total mass concentration of WSOM (c_{WSOM}) and NO_3^- ($c_{\mathrm{NO_3^-}}$).

$$f_{\mathrm{WSOM}} = \frac{c_{\mathrm{WSOM}}}{c_{\mathrm{NO_3^-}} + c_{\mathrm{WSOM}}} \quad \text{(S13)}$$

c_{WSOM} is derived from the GEOS-Chem model simulations, which use the simple scheme parameterization, as described in Pai *et al.* (*93*). In this scheme, WSOM from primary organic emission, including combustions from BB, residential fuels, and fossil fuels, is considered as water-soluble BrC (*77, 94*).

6.2 Global renoxification rates of HONO and NO_x based on the model simulations Using laboratory-based $J_{NO_3^- \rightarrow gas\_species}$ from Eqs. S11–S12, renoxification rates ($P_{NO_3^- \rightarrow gas\_species}$,

ppt hr^{-1}) were calculated by multiplying the $J_{NO_3^- \rightarrow gas\_species}$ with nitrate concentrations ($C_{NO_3^-}$) simulated by GEOS-Chem:

$$P_{NO_3^- \rightarrow gas\_species} = J_{NO_3^- \rightarrow gas\_species} \times C_{NO_3^-} \times 3600 \times 1000 \quad \text{(S14)}$$

$$P_{NO_3^- \rightarrow total} = J_{NO_3^- \rightarrow total} \times C_{NO_3^-} \times 3600 \times 1000 \quad \text{(S15)}$$

where 3600 converts seconds to hours, and 1000 converts from ppb to ppt. The annual mean $P_{NO_3^- \rightarrow gas\_species}$ in Fig. 5 is derived by multiplying the monthly mean surface-level $J_{NO_3^- \rightarrow gas\_species}$ with the monthly mean surface-level $C_{NO_3^-}$ and then averaging the resulting values annually.

7. Comparison of the laboratory-derived parameterization with in-plume wildfire observations

To assess the laboratory-derived parameterization against atmospheric observations, we compared it with the airborne wildfire measurements of Peng *et al.* (*11*) from the WE-CAN campaign (Western Wildfire Experiment for Cloud Chemistry, Aerosol Absorption and Nitrogen; NCAR C-130, western United States, summer 2018). We used their publicly archived per-transect box-model input dataset, which reports, for each plume transect, the aerosol nitrate and organic aerosol concentrations, the NO_2 photolysis frequency J_{NO_2}, the plume physical age, and the secondary HONO source inferred from closing the in-plume HONO budget.

For each transect, the water-soluble organic mass was estimated from the measured organic aerosol using the water-soluble fraction reported for WE-CAN brown carbon (*95*), and f_{WSOM} was computed as the ratio of the water-soluble organic mass to the sum of the water-soluble organic mass and aerosol nitrate (Eq. S13). The renoxification rate coefficient was then evaluated from Eq. S11 for two cases: with brown carbon, using the transect f_{WSOM}; and without brown carbon, setting $f_{WSOM} = 0$ so that only the direct nitrate photolysis term remains. In both cases the coefficient was scaled to the ambient light intensity of each transect through the factor $J_{NO_2}/J_{NO_2}^{exp}$. Multiplying the rate coefficient by the measured aerosol nitrate (Eq. S14) gives the predicted with-brown-carbon and without-brown-carbon HONO production for direct comparison with the inferred secondary source.

Including brown carbon increases the predicted HONO production by a factor of 8.2 ± 0.1 relative to nitrate photolysis alone. The predicted production remains below the inferred secondary

source in both cases, but the discrepancy decreases from a factor of approximately 700 without brown carbon to approximately 86 with brown carbon (fig. S10). The predicted production tracks the inferred secondary HONO source more closely for aged plumes (physical age > 3 h; Spearman $\rho = 0.81$, $n = 87$) than for fresh plumes (< 3 h; $\rho = 0.47$, $n = 95$), consistent with the proposed pathway acting as a secondary HONO source that develops with photochemical aging. The comparison is a process-level observational benchmark, and indicates that the parameterization provides a conservative estimate of this pathway under wildfire conditions.

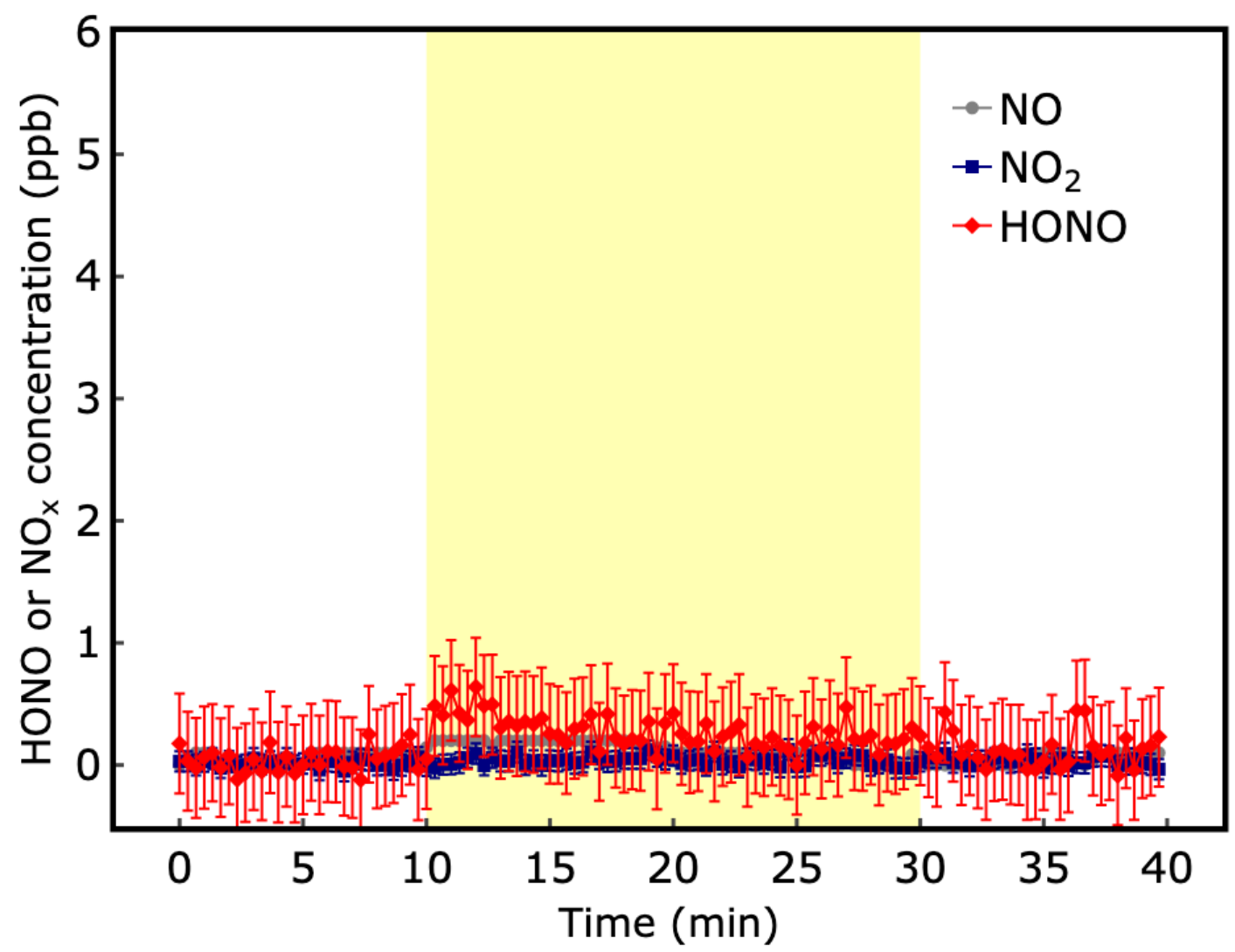

Fig. S1. Renoxification on a BrC sample without nitrate. Time profile of gas-phase HONO, NO_2 and NO concentrations during UV irradiation (> 310 nm) for the BrC blank sample (BrC + $(NH_4)_2SO_4$ + NH_4HSO_4). More details on the initial and equilibrium composition of the sample are listed in table S1. The experiments were conducted at an RH of 62% and a temperature of 296 K. Error bars represent the instrument uncertainties.

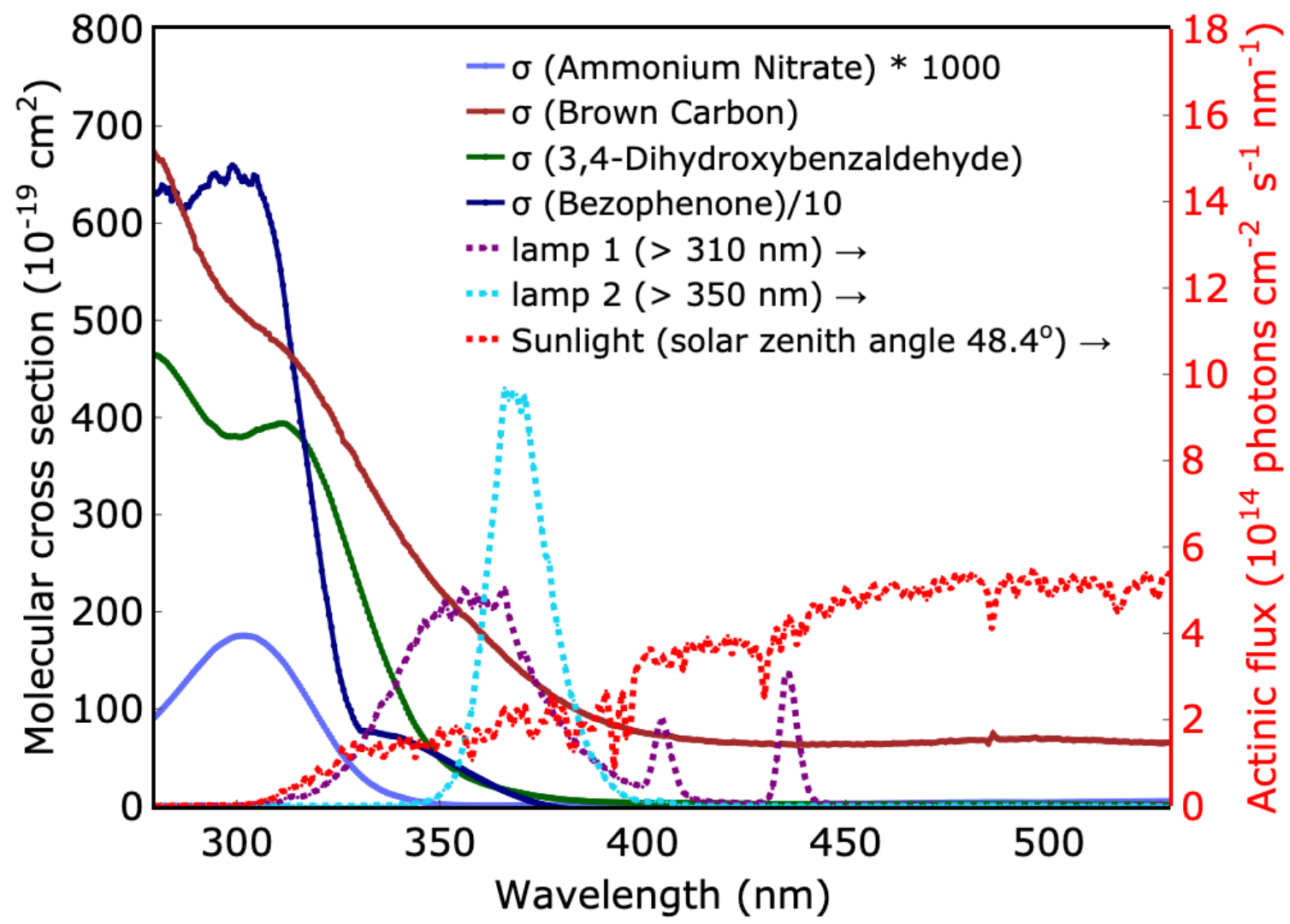

Fig. S2. Absorption spectra of different samples and irradiance profiles of light sources. The left y-axis represents the molecular cross-section (σ) of ammonium nitrate (scaled by a factor of 1000), the BrC sample I, 3,4-dihydroxybenzaldehyde (the DHB sample I), and benzophenone (the BP sample, scaled by a factor of 1/10). The light absorption of all solutions was measured at their raw pH, without additives. The right y-axis represents the spectral output of the two lamps used in the study and the solar irradiance spectra at 48° zenith angle.

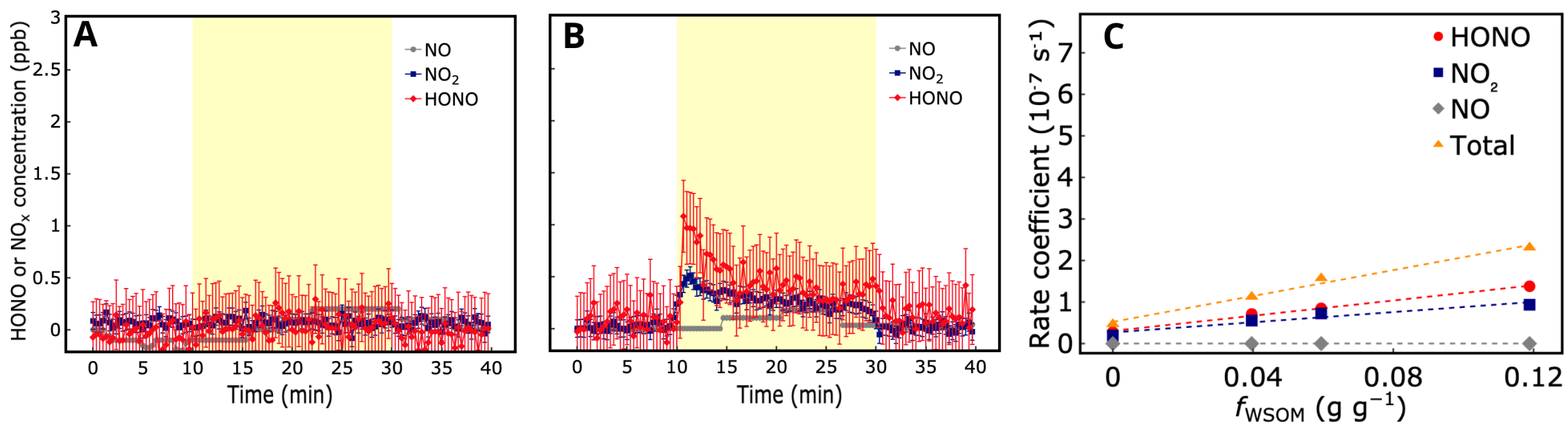

Fig. S3. Influence of BrC on renoxification during UV irradiation (> 350 nm). Time profile of gas-phase HONO, NO_2 and NO concentrations during UV irradiation (> 350 nm) for **(A)** the nitrate blank sample II (NH_4NO_3 + $(NH_4)_2SO_4$ + NH_4HSO_4) and **(B)** the BrC sample IV (BrC + NH_4NO_3 + $(NH_4)_2SO_4$ + NH_4HSO_4). The yellow region denotes the period of irradiation. **(C)** The rate coefficients for the photochemical renoxification of nitrate, leading to the production of gas species (HONO, NO_2 and NO) and the total rate coefficient, representing their sum, as a function of f_{WSOM} (the WSOM mass fraction relative to the total mass of NO_3^- and WSOM, g g^{-1}) of the BrC samples IV–VI and the nitrate blank sample II. The lines represent linear fits. More details on the initial and equilibrium composition of the samples are listed in table S1, and the f_{WSOM} calculation can be found in section S2. The experiments were conducted at an RH of 62% and a temperature of 296 K. Error bars represent the instrument uncertainties.

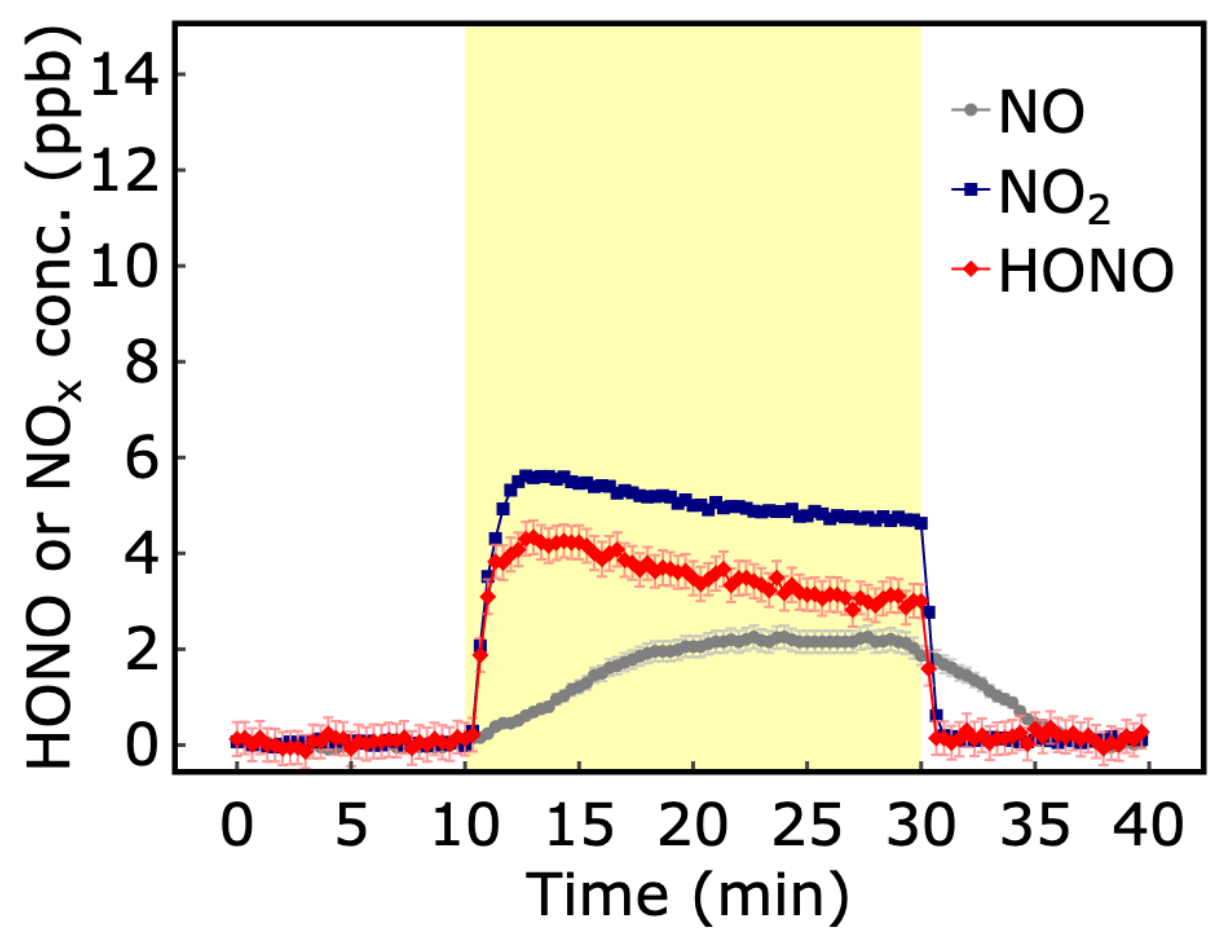

Fig. S4. Renoxification on a nitrate blank sample. Time profile of gas-phase HONO, NO_2 and NO concentrations during UV irradiation (> 310 nm) for the nitrate blank sample III (NH_4NO_3 + $(NH_4)_2SO_4$ + NH_4HSO_4). More details on the initial and equilibrium composition of the sample are listed in table S1. The experiments were conducted at an RH of 82% and a temperature of 291 K. Error bars represent the instrument uncertainties. Note that there is a difference in HONO and NO_x production between the nitrate blank samples I and III in Fig. 1B in the main text and fig. S4 here. The initial concentrations of NH_4NO_3 in the nitrate blank samples I and III are 0.01 M and 0.20 M, respectively, meaning that the total amount of NH_4NO_3 in the coated film of sample I is 20 times lower. As a result, HONO and NO_x production in sample I is negligible and falls below the detection limit. Therefore, when assessing the role of nitrate in renoxification in Fig. 3 of the main text, we used higher nitrate concentrations to ensure detectable levels of HONO and NO_x production.

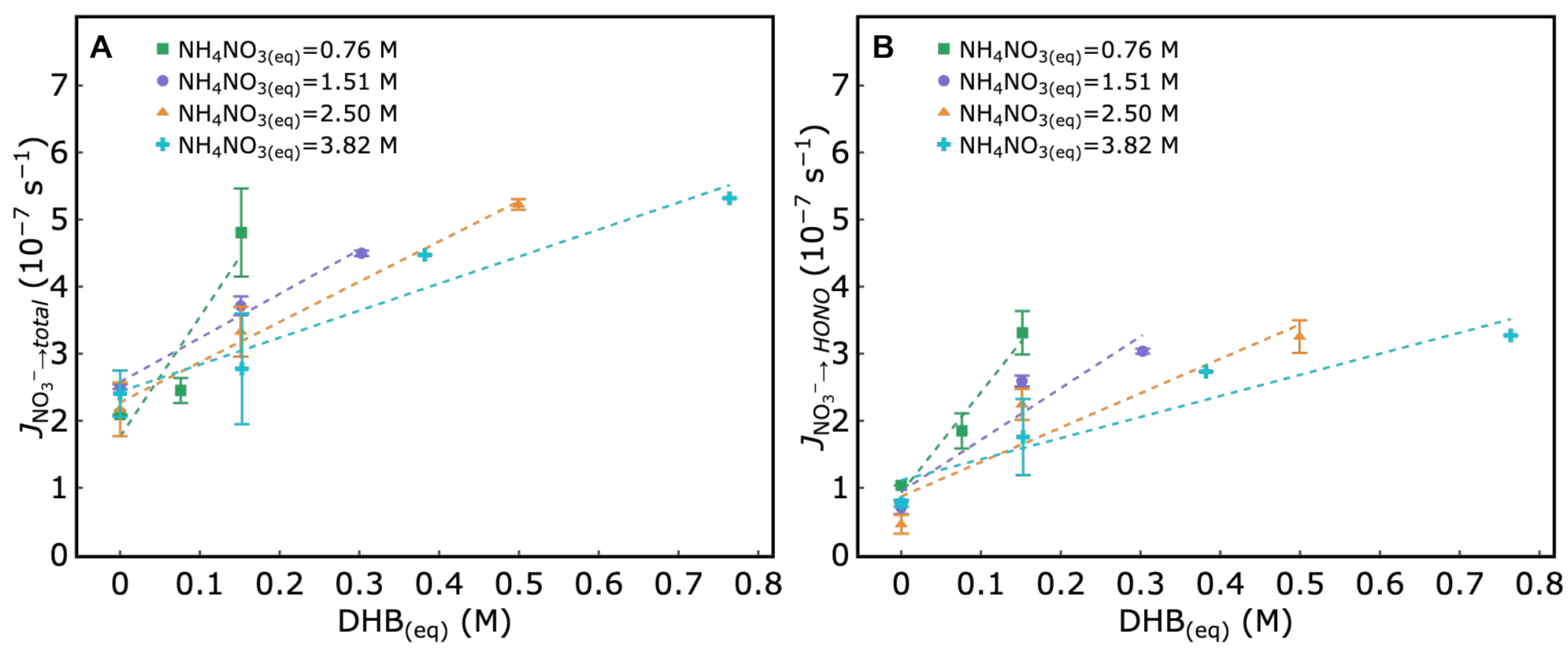

Fig. S5. Dependence of renoxification rate coefficients on DHB concentration under varying nitrate conditions. (A) $J_{NO_3^- \to total}$ and (B) $J_{NO_3^- \to HONO}$ are shown as functions of the equilibrium DHB concentration. Different colors represent samples at different equilibrium concentrations of NH_4NO_3, as indicated in the legend. Dashed lines show linear regressions for data obtained at the same nitrate concentration and are included to guide the eye. More details on the initial and equilibrium composition of the samples are provided in table S1. Error bars denote standard deviations from duplicate experiments.

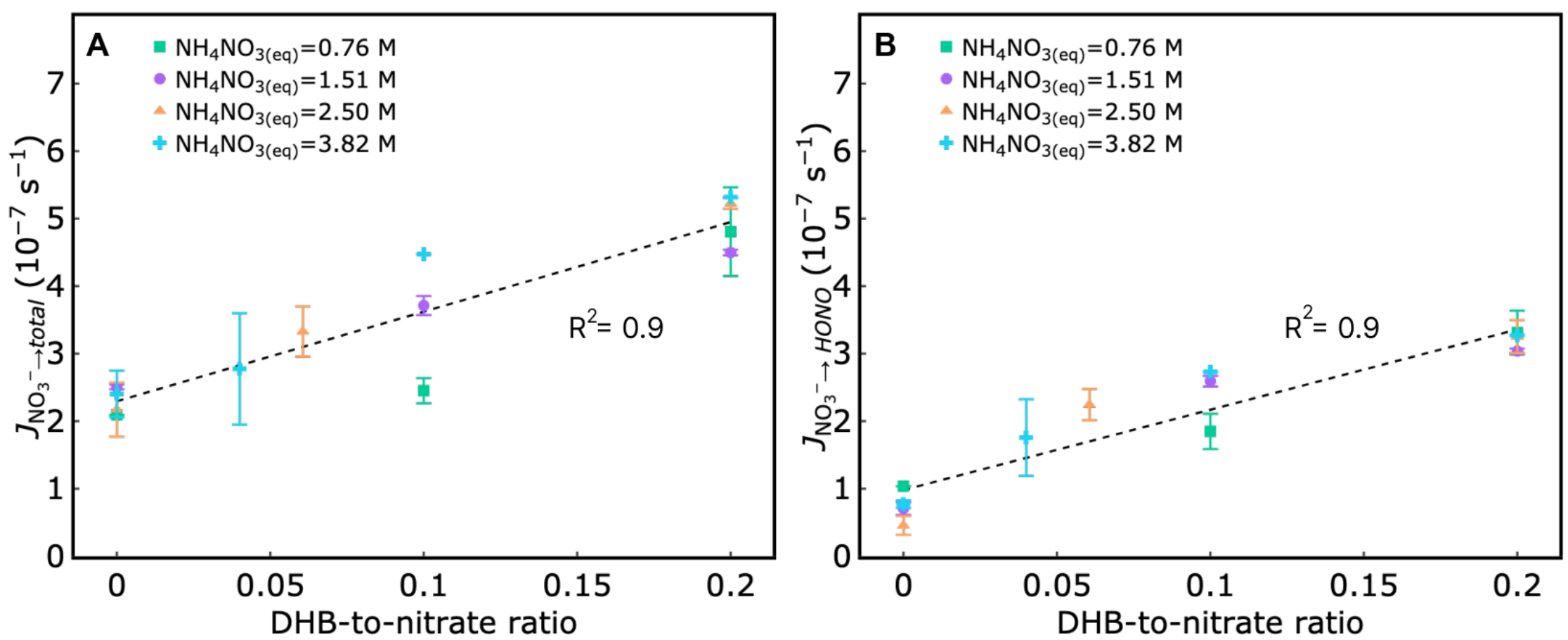

Fig. S6. Dependence of renoxification rate coefficients on the DHB-to-nitrate ratio under varying nitrate conditions. (A) $J_{NO_3^- \rightarrow total}$ and (B) $J_{NO_3^- \rightarrow HONO}$ are shown as functions of the molar DHB-to-nitrate ratio, with different colors representing samples at different equilibrium concentrations of NH_4NO_3, as indicated in the legend. Dashed lines represent linear fits. More details on the initial and equilibrium composition of the samples are provided in table S1. Error bars denote standard deviations from duplicate experiments.

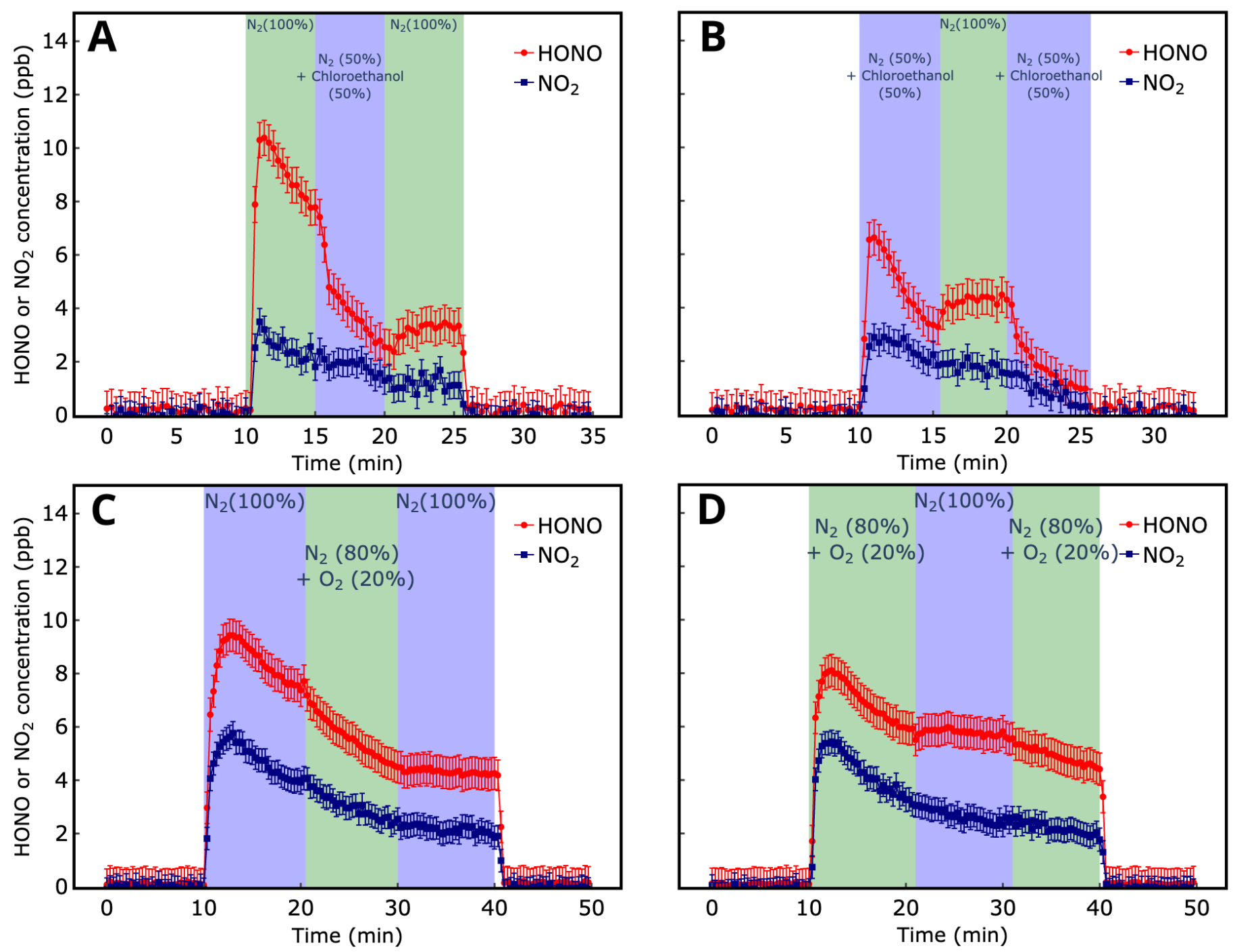

Fig. S7. Influence of electron scavenger on renoxification. Time profiles of gas-phase HONO and NO_2 concentrations during UV irradiation (>310 nm) for DHB samples X–XIII (DHB + NH_4NO_3 + $(NH_4)_2SO_4$ + NH_4HSO_4) under varying gas flow conditions. The conditions include: 100% N_2; a mixed gas flow of N_2 (50%) and chloroethanol vapor (50%); and a mixed gas flow of N_2 (80%) and O_2 (20%), with periodic switching between these conditions. The specific gas flow conditions are annotated in the figure. The experiments were conducted at an RH of 42% and a temperature of 296 K. Error bars represent the instrument uncertainties.

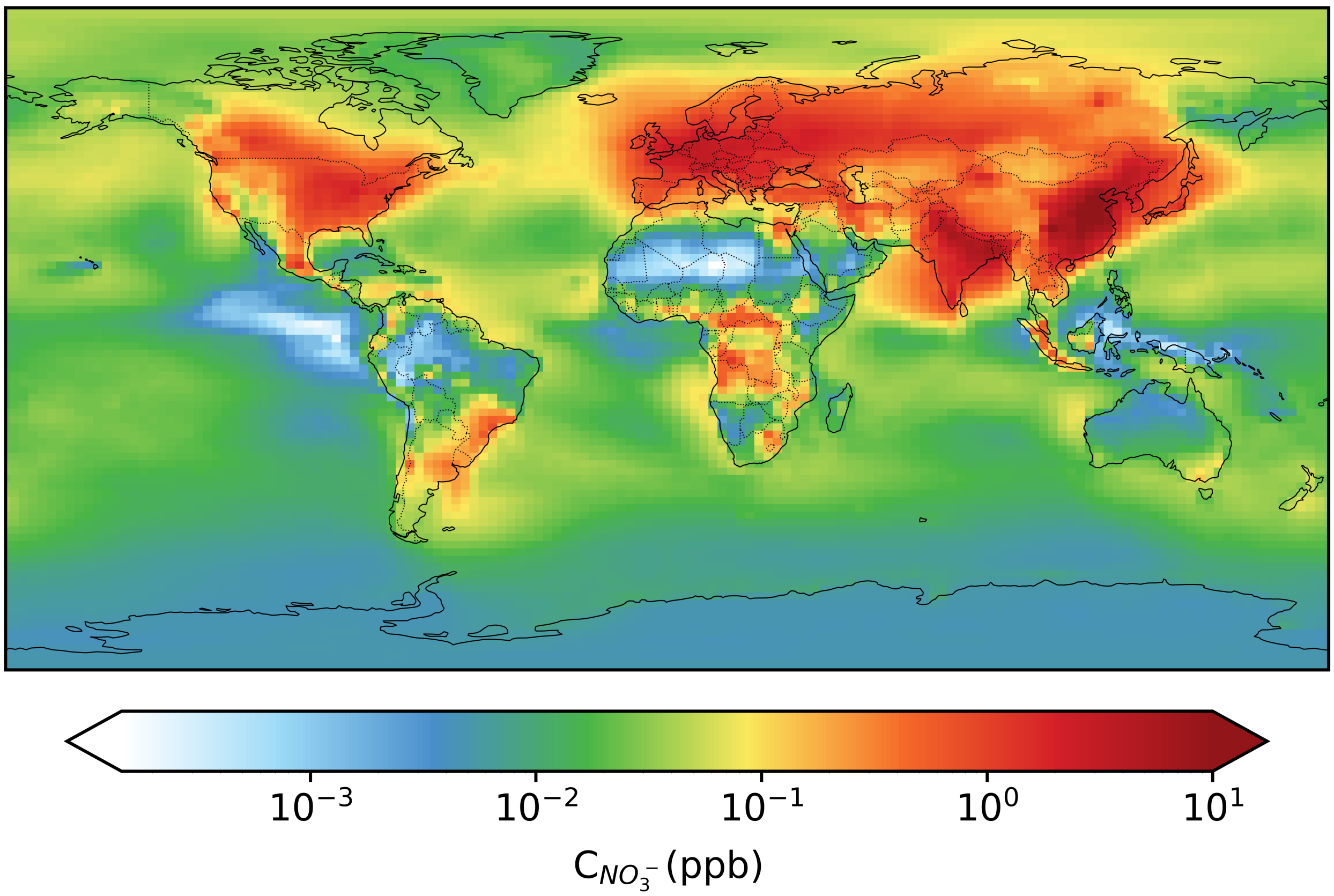

Fig. S8. Global distributions of surface-level annual mean nitrate concentration in aerosols. The surface-level annual mean nitrate concentration in aerosols ($C_{NO_3^-}$, ppb) is derived by averaging the monthly mean values simulated using GEOS-Chem, as detailed in section S6.

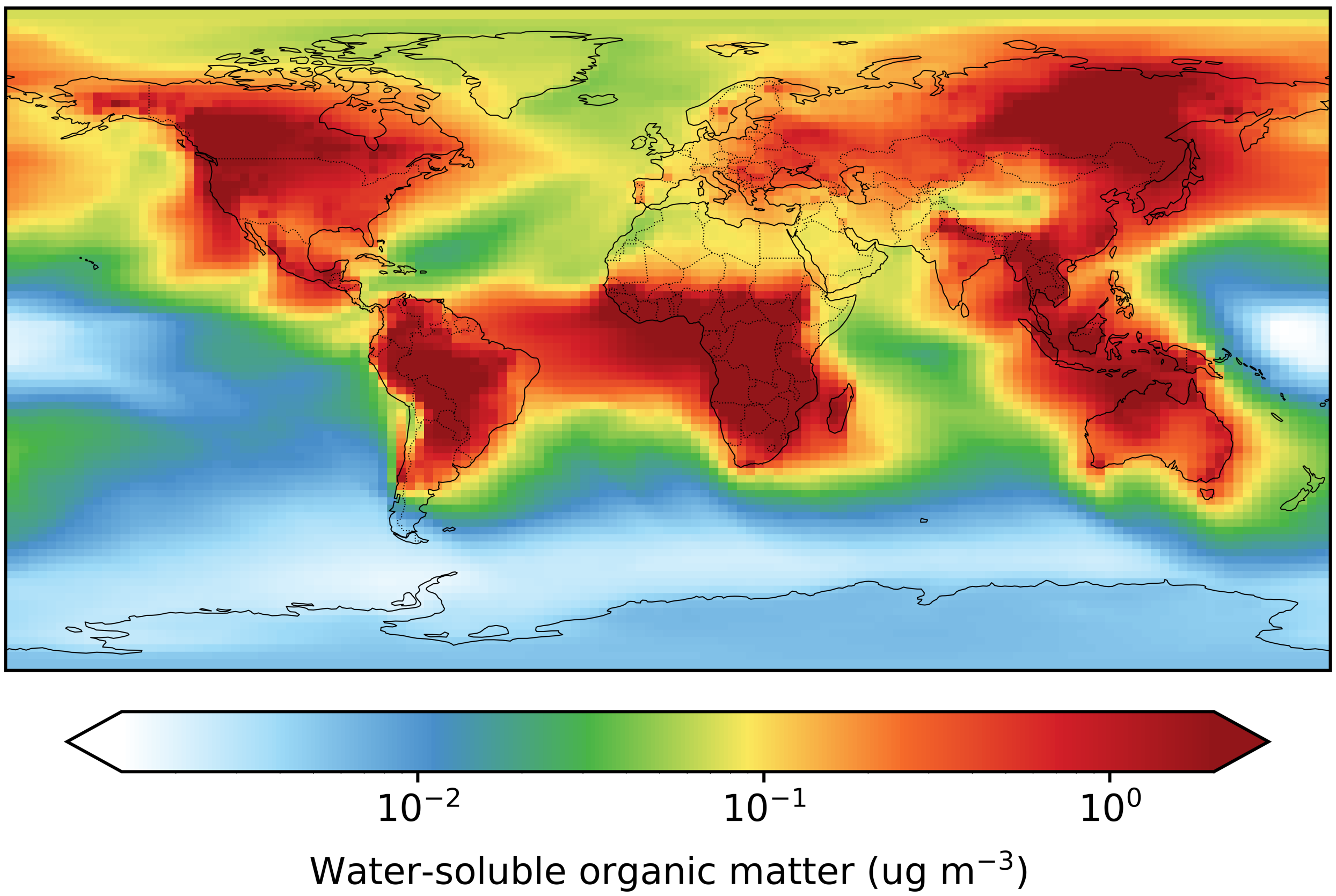

Fig. S9. Global distributions of WSOM in aerosols. The surface-level WSOM (μg m^{-3}) is derived by averaging the monthly mean values simulated using GEOS-Chem, as detailed in section S6.

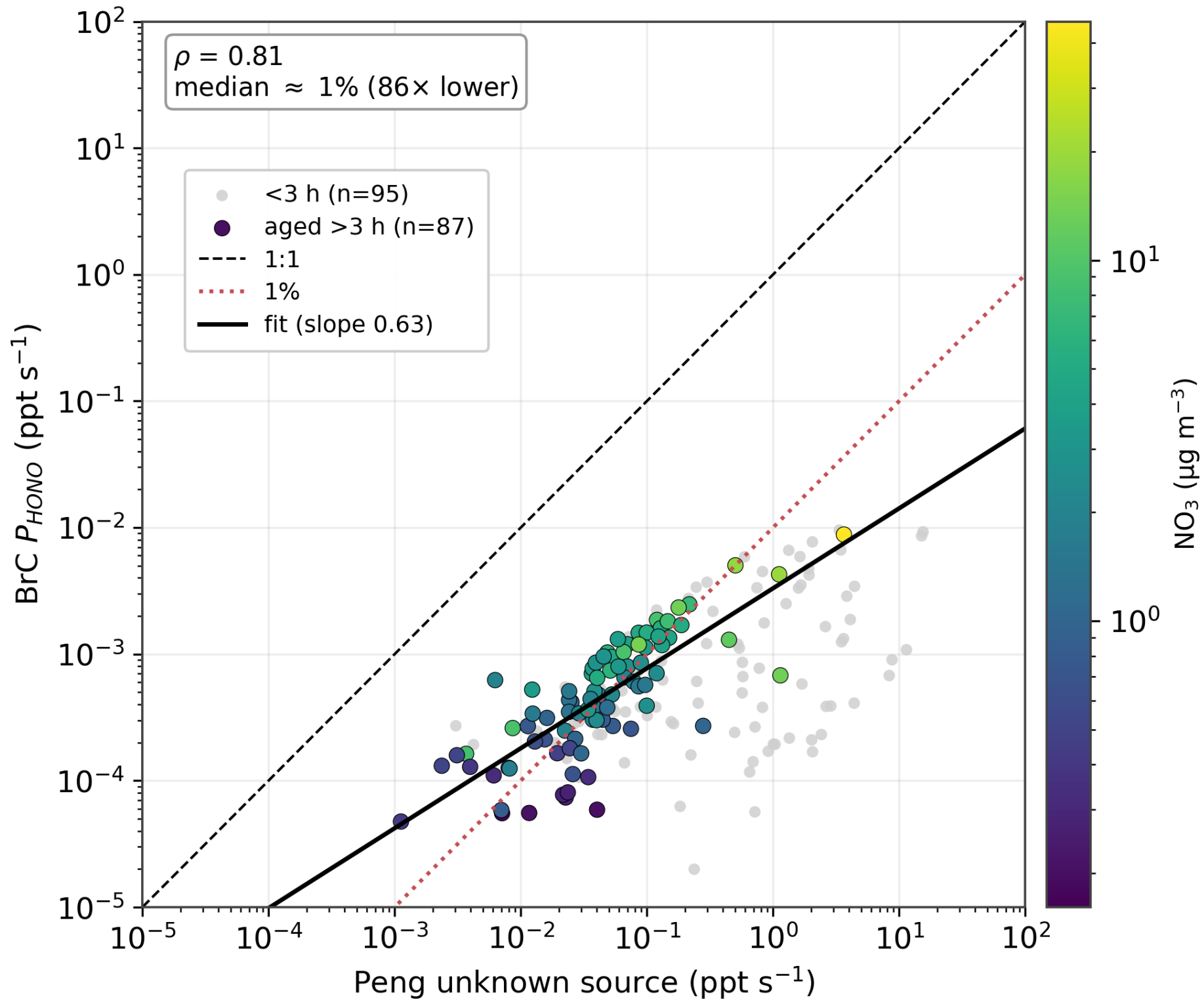

Fig. S10. Predicted BrC-driven HONO production compared, per WE-CAN 2018 wildfire plume transect, with the secondary HONO source inferred by Peng *et al.* (*11*), computed here by applying their steady-state HONO budget to the per-transect data. Grey symbols, fresh plumes ($<$ 3 h, n = 95); coloured symbols, aged plumes ($>$ 3 h, n = 87), shaded by aerosol nitrate. Dashed line, 1:1; dotted line, 1% of the inferred secondary HONO source; solid line, least-squares fit to the aged transects in log-log space (slope 0.63). For aged transects the predicted production is a median 1.2% of the inferred secondary HONO source, an offset of about 86 times, with Spearman $\rho = 0.81$. The calculation is described in section S7. WE-CAN measurements are from the NSF NCAR Earth Observing Laboratory archive: HR-ToF-AMS (*96*), PILS1 water-soluble organic carbon (*97*), and HARP photolysis frequencies (*98*); per-transect plume data are from Peng *et al.* (*99*).

Table S1. Initial and equilibrium sample concentrations with experimental conditions for various samples

Sample name	Relevant Figure	Initial sample concentration (M)				Equilibrium sample concentration (M)				Equilibrium condition				
		WSOM	NH_4NO_3	$(NH_4)_2SO_4$	$NH_4HSO_4 \times 1000$	WSOM	NO_3^-	SO_4^{2-}	HSO_4^-	pH	Volume (μL)	Lamp	RH (%)	Temp (K)
BrC I	Figs. 1A, 1C and 4	0.42	0.01	0.49	0.49	5.05	0.12	5.96	0.0051	3.75	24.87	>310 nm	62	296
BrC II	Figs. 1A, 1C and 4	0.21	0.01	0.49	0.49	2.52	0.12	5.96	0.0051	3.75	24.87	>310 nm	62	296
BrC III	Figs. 1A, 1C and 4	0.14	0.01	0.49	0.49	1.68	0.12	5.96	0.0051	3.75	24.87	>310 nm	62	296
Nitrate blank I	Figs 1B and 4	0.42	0.01	0.49	0.49	5.05	0.12	5.96	0.0051	3.75	24.87	>310 nm	62	296
Nitrate blank II	Figure S3A	0.42	0.01	0.49	0.49	5.05	0.12	5.96	0.0051	3.75	24.87	>350 nm	62	296
DHB I	Figure 2B	0.02	0.20	0.37	0.37	0.15	1.51	2.81	0.0022	3.83	39.64	>310 nm	82	291
BP	Figure 2C	0.02	0.20	0.37	0.37	0.15	1.51	2.81	0.0022	3.83	39.64	>310 nm	82	291
Nitrate Blank III	Figs. 3A, 3D, 4 and S4-S6	0.00	0.20	0.37	0.37	0.00	1.51	2.81	0.0022	3.83	39.64	>310 nm	82	291
Nitrate Blank IV	Figs. 3A, 3D, 4 and S5-S6	0.00	0.10	0.44	0.44	0.00	0.76	3.31	0.0025	3.78	39.49	>310 nm	82	291
Nitrate Blank V	Figs. 3A, 3D, 4 and S5-S6	0.00	0.33	0.29	0.29	0.00	2.5	2.18	0.0018	3.90	39.64	>310 nm	82	291
Nitrate Blank VI	Figs. 3A, 3D, 4 and S5-S6	0.00	0.50	0.18	0.18	0.00	3.82	1.37	0.0012	3.99	39.26	>310 nm	82	291
DHB II	Figs. 3B, 3E, 4 and S5-S6	0.02	0.10	0.44	0.44	0.15	0.76	3.31	0.0025	3.78	39.49	>310 nm	82	291
DHB III	Figs. 3B, 3E, 4 and S5-S6	0.04	0.20	0.37	0.37	0.30	1.51	2.81	0.0022	3.83	39.64	>310 nm	82	291
DHB IV	Figs. 3B, 3E, 4 and S5-S6	0.07	0.33	0.29	0.29	0.50	2.5	2.18	0.0018	3.90	39.64	>310 nm	82	291
DHB V	Figs. 3B, 3E, 4 and S5-S6	0.10	0.50	0.18	0.18	0.76	3.82	1.37	0.0012	3.99	39.26	>310 nm	82	291
DHB VI	Figs. 3C, 3F, 4 and S5-S6	0.02	0.10	0.44	0.44	0.15	0.76	3.31	0.0025	3.78	39.49	>310 nm	82	291
DHB VII	Figs. 3C, 3F, 4 and S5-S6	0.02	0.20	0.37	0.37	0.15	1.51	2.81	0.0022	3.83	39.64	>310 nm	82	291
DHB VIII	Figs. 3C, 3F, 4 and S5-S6	0.02	0.33	0.29	0.29	0.15	2.5	2.18	0.0018	3.90	39.64	>310 nm	82	291
DHB IX	Figs. 3C, 3F, 4 and S5-S6	0.02	0.50	0.18	0.18	0.15	3.82	1.37	0.0012	3.99	39.26	>310 nm	82	291
BrC IV	Fig. S3B and Fig. S3C	0.42	0.01	0.49	0.49	5.05	0.12	5.96	0.0051	3.75	24.87	>350 nm	62	296
BrC V	Fig. S3C	0.21	0.01	0.49	0.49	2.52	0.12	5.96	0.0051	3.75	24.87	>350 nm	62	296
BrC VI	Fig. S3C	0.14	0.01	0.49	0.49	1.68	0.12	5.96	0.0051	3.75	24.87	>350 nm	62	296
BrC blank	Fig. S1	0.42	0.00	0.49	0.49	5.05	0.12	5.96	0.0051	3.75	24.87	>310 nm	62	296
DHB X, XI, XII, XIII	Fig. S7A–D	0.42	0.01	0.49	0.49	0.06	0.12	5.96	0.0051	3.75	24.87	>310 nm	42	296

REFERENCES

1. M. Kulmala, T. Petäjä, Soil nitrites influence atmospheric chemistry. *Science* **333**, 1586–1587 (2011).

2. R. C. Chapleski, Y. Zhang, D. Troya, J. R. Morris, Heterogeneous chemistry and reaction dynamics of the atmospheric oxidants, O_3, NO_3, and OH, on organic surfaces. *Chem. Soc. Rev.* **45**, 3731–3746 (2016).

3. V. Shah, D. J. Jacob, R. Dang, L. N. Lamsal, S. A. Strode, S. D. Steenrod, K. F. Boersma, S. D. Eastham, T. M. Fritz, C. Thompson, J. Peischl, I. Bourgeois, I. B. Pollack, B. A. Nault, R. C. Cohen, P. Campuzano-Jost, J. L. Jimenez, S. T. Andersen, L. J. Carpenter, T. Sherwen, M. J. Evans, Nitrogen oxides in the free troposphere: Implications for tropospheric oxidants and the interpretation of satellite NO_2 measurements. *Atmos. Chem. Phys.* **23**, 1227–1257 (2023).

4. P. Kasibhatla, T. Sherwen, M. J. Evans, L. J. Carpenter, C. Reed, B. Alexander, Q. Chen, M. P. Sulprizio, J. D. Lee, K. A. Read, W. Bloss, L. R. Crilley, W. C. Keene, A. A. P. Pszenny, A. Hodzic, Global impact of nitrate photolysis in sea-salt aerosol on NO_x, OH, and O_3 in the marine boundary layer. *Atmos. Chem. Phys.* **18**, 11185–11203 (2018).

5. J. Lelieveld, J. S. Evans, M. Fnais, D. Giannadaki, A. Pozzer, The contribution of outdoor air pollution sources to premature mortality on a global scale. *Nature* **525**, 367–371 (2015).

6. H. Su, Y. F. Cheng, M. Shao, D. F. Gao, Z. Y. Yu, L. M. Zeng, J. Slanina, Y. H. Zhang, A. Wiedensohler, Nitrous acid (HONO) and its daytime sources at a rural site during the 2004 PRIDE-PRD experiment in China. *J. Geophys. Res.* **113**, D14312 (2008).

7. Z. Tan, H. Fuchs, K. Lu, A. Hofzumahaus, B. Bohn, S. Broch, H. Dong, S. Gomm, R. Häseler, L. He, F. Holland, X. Li, Y. Liu, S. Lu, F. Rohrer, M. Shao, B. Wang, M. Wang, Y. Wu, L. Zeng, Y. Zhang, A. Wahner, Y. Zhang, Radical chemistry at a rural site (Wangdu) in the North China Plain: Observation and model calculations of OH, HO_2 and RO_2 radicals. *Atmos. Chem. Phys.* **17**, 663–690 (2017).

8. K. Acker, A. Febo, S. Trick, C. Perrino, P. Bruno, P. Wiesen, D. Möller, W. Wieprecht, R. Auel, M. Giusto, A. Geyer, U. Platt, I. Allegrini, Nitrous acid in the urban area of Rome. *Atmos. Environ.* **40**, 3123–3133 (2006).

9. C. D. Fredrickson, N. Theys, J. A. Thornton, Satellite evidence of HONO/NO_2 increase with fire radiative power. *Geophys. Res. Lett.* **50**, e2022GL101707 (2023).

10. R. Gu, P. Zheng, T. Chen, C. Dong, Y. Wang, Y. Liu, Y. Liu, Y. Luo, G. Han, X. Wang, X. Zhou, T. Wang, W. Wang, L. Xue, Atmospheric nitrous acid (HONO) at a rural coastal site in North China: Seasonal variations and effects of biomass burning. *Atmos. Environ.* **229**, 117429 (2020).

11. Q. Peng, B. B. Palm, C. D. Fredrickson, B. H. Lee, S. R. Hall, K. Ullmann, A. J. Weinheimer, E. Levin, P. De Mott, L. A. Garofalo, M. A. Pothier, D. K. Farmer, E. V. Fischer, J. A. Thornton, Direct constraints on secondary HONO production in aged wildfire smoke from airborne measurements over the western US. *Geophys. Res. Lett.* **49**, e2021GL097609 (2022).

12. N. Theys, R. Volkamer, J.-F. Müller, K. J. Zarzana, N. Kille, L. Clarisse, I. De Smedt, C. Lerot, H. Finkenzeller, F. Hendrick, T. K. Koenig, C. F. Lee, C. Knote, H. Yu, M. Van Roozendael, Global nitrous acid emissions and levels of regional oxidants enhanced by wildfires. *Nat. Geosci.* **13**, 681–686 (2020).

13. B. Franco, L. Clarisse, N. Theys, J. Hadji-Lazaro, C. Clerbaux, P. Coheur, Pyrogenic HONO seen from space: Insights from global IASI observations. *Atmos. Chem. Phys.* **23**, 4973–5007 (2023).

14. M. Keywood, M. Kanakidou, A. Stohl, F. Dentener, G. Grassi, C. P. Meyer, K. Torseth, D. Edwards, A. M. Thompson, U. Lohmann, J. Burrows, Fire in the air: Biomass burning impacts in a changing climate. *Crit. Rev. Environ. Sci. Technol.* **43**, 40–83 (2013).

15. J. T. Abatzoglou, A. P. Williams, Impact of anthropogenic climate change on wildfire across western US forests. *Proc. Natl. Acad. Sci. U.S.A.* **113**, 11770–11775 (2016).

16. J. K. Balch, J. T. Abatzoglou, M. B. Joseph, M. J. Koontz, A. L. Mahood, J. M. Glinchy, M. E. Cattau, A. P. Williams, Warming weakens the night-time barrier to global fire. *Nature* **602**, 442–448 (2022).

17. H. K. Pullabhotla, M. Zahid, S. Heft-Neal, V. Rathi, M. Burke, Global biomass fires and infant mortality. *Proc. Natl. Acad. Sci. U.S.A.* **120**, e2218210120 (2023).

18. O. Pechony, D. T. Shindell, Driving forces of global wildfires over the past millennium and the forthcoming century. *Proc. Natl. Acad. Sci. U.S.A.* **107**, 19167–19170 (2010).

19. C. Wu, S. Venevsky, S. Sitch, L. M. Mercado, C. Huntingford, A. C. Staver, Historical and future global burned area with changing climate and human demography. *One Earth* **4**, 517–530 (2021).

20. Q. Peng, B. B. Palm, K. E. Melander, B. H. Lee, S. R. Hall, K. Ullmann, T. Campos, A. J. Weinheimer, E. C. Apel, R. S. Hornbrook, A. J. Hills, D. D. Montzka, F. Flocke, L. Hu, W. Permar, C. Wielgasz, J. Lindaas, I. B. Pollack, E. V. Fischer, T. H. Bertram, J. A. Thornton, HONO emissions from western US wildfires provide dominant radical source in fresh wildfire smoke. *Environ. Sci. Technol.* **54**, 5954–5963 (2020).

21. J. Chai, J. E. Dibb, B. E. Anderson, C. Bekker, D. E. Blum, E. Heim, C. E. Jordan, E. E. Joyce, J. H. Kaspari, H. Munro, W. W. Walters, M. G. Hastings, Isotopic evidence for dominant secondary production of HONO in near-ground wildfire plumes. *Atmos. Chem. Phys.* **21**, 13077–13098 (2021).

22. Q. Peng, B. B. Palm, C. D. Fredrickson, B. H. Lee, S. R. Hall, K. Ullmann, T. Campos, A. J. Weinheimer, E. C. Apel, F. Flocke, W. Permar, L. Hu, L. A. Garofalo, M. A. Pothier, D. K. Farmer, I.-T. Ku, A. P. Sullivan, J. L. Collett, E. F. Jr, J. A. Thornton, Observations and modeling of NO_x photochemistry and fate in fresh wildfire plumes. *ACS Earth Space Chem.* **5**, 2652–2667 (2021).

23. C. Ye, X. Zhou, D. Pu, J. Stutz, J. Festa, M. Spolaor, C. Tsai, C. Cantrell, R. L. Mauldin III, T. Campos, A. Weinheimer, R. S. Hornbrook, E. C. Apel, A. Guenther, L. Kaser, B. Yuan, T.

Karl, J. Haggerty, S. Hall, K. Ullmann, J. N. Smith, J. Ortega, C. Knote, Rapid cycling of reactive nitrogen in the marine boundary layer. *Nature* **532**, 489–491 (2016).

24. R. Sommariva, M. S. Alam, L. R. Crilley, D. J. Rooney, W. J. Bloss, K. W. Fomba, S. T. Andersen, L. J. Carpenter, Factors influencing the formation of nitrous acid from photolysis of particulate nitrate. *J. Phys. Chem. A* **127**, 9302–9310 (2023).

25. B. Li, J. Gao, C. Chen, L. Wen, Y. Zhang, J. Li, Y. Zhang, X. Du, K. Zhang, J. Wang, Exploring HONO production from particulate nitrate photolysis in representative regions of China: Characteristics, influencing factors, and environmental implications. *Atmos. Chem. Phys.* **24**, 13183–13198 (2024).

26. S. T. Andersen, L. J. Carpenter, C. Reed, J. D. Lee, R. Chance, T. Sherwen, A. R. Vaughan, J. Stewart, P. M. Edwards, W. J. Bloss, R. Sommariva, L. R. Crilley, G. J. Nott, L. Neves, K. Read, D. E. Heard, P. W. Seakins, L. K. Whalley, G. A. Boustead, L. T. Fleming, D. Stone, K. W. Fomba, Extensive field evidence for the release of HONO from the photolysis of nitrate aerosols. *Sci. Adv.* **9**, eadd6266 (2023).

27. C. Ye, N. Zhang, H. Gao, X. Zhou, Photolysis of particulate nitrate as a source of HONO and NO_x. *Environ. Sci. Technol.* **51**, 6849–6856 (2017).

28. C. Ye, H. Gao, N. Zhang, X. Zhou, Photolysis of nitric acid and nitrate on natural and artificial surfaces. *Environ. Sci. Technol.* **50**, 3530–3536 (2016).

29. C. Ye, N. Zhang, H. Gao, X. Zhou, Matrix effect on surface-catalyzed photolysis of nitric acid. *Sci. Rep.* **9**, 14875 (2019).

30. X. Zhang, Q. Huang, Y.-X. Liu, J. Yin, S.-F. Pang, P. Liu, Y.-H. Zhang, M. Ge, Microdroplet surface drives and accelerates proton-controlled, size-dependent nitrate photolysis. *J. Am. Chem. Soc.* **147**, 19595–19601 (2025).

31. J. J. Jankowski, D. J. Kieber, K. Mopper, P. J. Neale, Development and intercalibration of ultraviolet solar actinometers. *Photochem. Photobiol.* **71**, 431–440 (2000).

32. P. Nissenson, D. Dabdub, R. Das, V. Maurino, C. Minero, D. Vione, Evidence of the water-cage effect on the photolysis of NO_3^- and $FeOH^{2+}$. Implications of this effect and of H_2O_2 surface accumulation on photochemistry at the air-water interface of atmospheric droplets. *Atmos. Environ.* **44**, 4859–4866 (2010).

33. C. Zhu, B. Xiang, L. Zhu, R. Cole, Determination of absorption cross sections of surface-adsorbed HNO_3 in the 290–330 nm region by Brewster angle cavity ring-down spectroscopy. *Chem. Phys. Lett.* **458**, 373–377 (2008).

34. J. Du, L. Zhu, Quantification of the absorption cross sections of surface-adsorbed nitric acid in the 335-365 nm region by Brewster angle cavity ring-down spectroscopy. *Chem. Phys. Lett.* **511**, 213–218 (2011).

35. M. Sangwan, W. R. Stockwell, D. Stewart, L. Zhu, Absorption of near UV light by HNO_3/NO_3^- on sapphire surfaces. *J. Phys. Chem. A* **120**, 2877–2884 (2016).

36. D. E. Otten, P. B. Petersen, R. J. Saykally, Observation of nitrate ions at the air/water interface by UV-second harmonic generation. *Chem. Phys. Lett.* **449**, 261–265 (2007).

37. M. Roca, J. Zahardis, J. Bone, M. El-Maazawi, V. H. Grassian, 310 nm irradiation of atmospherically relevant concentrated aqueous nitrate solutions: Nitrite production and quantum yields. *J. Phys. Chem. A* **112**, 13275–13281 (2008).

38. P. K. Hudson, J. Schwarz, J. Baltrusaitis, E. R. Gibson, V. H. Grassian, A spectroscopic study of atmospherically relevant concentrated aqueous nitrate solutions. *J. Phys. Chem. A* **111**, 544–548 (2007).

39. S. Laufs, J. Kleffmann, Investigations on HONO formation from photolysis of adsorbed HNO_3 on quartz glass surfaces. *Phys. Chem. Chem. Phys.* **18**, 9616–9625 (2016).

40. L. M. Pegram, M. T. Record, Partitioning of atmospherically relevant ions between bulk water and the water/vapor interface. *Proc. Natl. Acad. Sci. U.S.A.* **103**, 14278–14281 (2006).

41. M. A. Brown, B. Winter, M. Faubel, J. C. Hemminger, Spatial distribution of nitrate and nitrite anions at the liquid/vapor interface of aqueous solutions. *J. Am. Chem. Soc.* **131**, 8354–8355 (2009).

42. Y. Wang, D. D. Huang, W. Huang, B. Liu, Q. Chen, R. Huang, M. Gen, B. R. Go, C. K. Chan, X. Li, T. Hao, Y. Tan, K. I. Hoi, K. M. Mok, Y. J. Li, Enhanced nitrite production from the aqueous photolysis of nitrate in the presence of vanillic acid and implications for the roles of light-absorbing organics. *Environ. Sci. Technol.* **55**, 15694–15704 (2021).

43. F. Bao, H. Jiang, Y. Zhang, M. Li, C. Ye, W. Wang, M. Ge, C. Chen, J. Zhao, The key role of sulfate in the photochemical renoxification on real $PM_{2.5}$. *Environ. Sci. Technol.* **54**, 3121–3128 (2020).

44. H. Jiang, F. Bao, J. Wang, J. Chen, Y. Zhu, D. Huang, C. Chen, J. Zhao, Direct formation of electronic excited NO_2 contributes to the high yield of HONO during photosensitized renoxification. *Environ. Sci. Technol.* **57**, 11144–11151 (2023).

45. S. L. M. Garcia, S. Pandit, J. G. Navea, V. H. Grassian, Nitrous acid (HONO) formation from the irradiation of aqueous nitrate solutions in the presence of marine chromophoric dissolved organic matter: Comparison to other organic photosensitizers. *ACS Earth Space Chem.* **5**, 3056–3064 (2021).

46. P. Kim, C. Boothby, V. H. Grassian, R. E. Continetti, Photoinduced reactions of nitrate in aqueous microdroplets by triplet energy transfer. *J. Phys. Chem. Lett.* **14**, 10677–10684 (2023).

47. Q. Shi, Y. Tao, J. E. Krechmer, C. L. Heald, J. G. Murphy, J. H. Kroll, Q. Ye, Laboratory investigation of renoxification from the photolysis of inorganic particulate nitrate. *Environ. Sci. Technol.* **55**, 854–861 (2021).

48. W. Jiang, M. V. Misovich, A. P. S. Hettiyadura, A. Laskin, A. S. Mc Fall, C. Anastasio, Q. Zhang, Photosensitized reactions of a phenolic carbonyl from wood combustion in the aqueous phase—Chemical evolution and light absorption properties of AqSOA. *Environ. Sci. Technol.* **55**, 5199–5211 (2021).

49. C. George, R. S. Strekowski, J. Kleffmann, K. Stemmler, M. Ammann, Photoenhanced uptake of gaseous NO_2 on solid organic compounds: A photochemical source of HONO? *Faraday Discuss.* **130**, 195–210 (2005).

50. P. C. Arroyo, T. Bartels-Rausch, P. A. Alpert, S. Dumas, S. Perrier, C. George, M. Ammann, Particle-phase photosensitized radical production and aerosol aging. *Environ. Sci. Technol.* **52**, 7680–7688 (2018).

51. K. Stemmler, M. Ammann, C. Donders, J. Kleffmann, C. George, Photosensitized reduction of nitrogen dioxide on humic acid as a source of nitrous acid. *Nature* **440**, 195–198 (2006).

52. S. Canonica, T. Kohn, M. Mac, F. J. Real, J. Wirz, U. von Gunten, Photosensitizer method to determine rate constants for the reaction of carbonate radical with organic compounds. *Environ. Sci. Technol.* **39**, 9182–9188 (2005).

53. L. I. Grossweiner, G. W. Swenson, E. F. Zwicker, Photochemical generation of the hydrated electron. *Science* **141**, 805–806 (1963).

54. R. G. Zepp, A. M. Braun, J. Hoigné, J. A. Leenheer, Photoproduction of hydrated electrons from natural organic solutes in aquatic environments. *Environ. Sci. Technol.* **21**, 485–490 (1987).

55. W. Wang, O. C. Zafiriou, I.-Y. Chan, R. G. Zepp, N. V. Blough, Production of hydrated electrons from photoionization of dissolved organic matter in natural waters. *Environ. Sci. Technol.* **41**, 1601–1607 (2007).

56. X. Chen, D. S. Larsen, S. E. Bradforth, I. H. M. van Stokkum, Broadband spectral probing revealing ultrafast photochemical branching after ultraviolet excitation of the aqueous phenolate anion. *J. Phys. Chem. A* **115**, 3807–3819 (2011).

57. A. Bussandri, H. van Willigen, Photoionization of phenolates and scavenging of hydrated electrons by NO_3^-: A study of the reaction mechanism by FT-EPR. *J. Phys. Chem. A* **105**, 4669–4675 (2001).

58. J. Feitelson, E. Hayon, Electron ejection and electron capture by phenolic compounds. *J. Phys. Chem.* **77**, 10–15 (1973).

59. D. V. Bent, E. Hayon, Excited state chemistry of aromatic amino acids and related peptides. I. Tyrosine. *J. Am. Chem. Soc.* **97**, 2599–2606 (1975).

60. A. R. Cook, N. Dimitrijevic, B. W. Dreyfus, D. Meisel, L. A. Curtiss, D. M. Camaioni, Argonne National Laboratory (United States), Reducing radicals in nitrate solutions. The NO_3^{2-} system revisited. *J. Phys. Chem. A* **105**, 3658–3666 (2001).

61. B. Abel, U. Buck, A. L. Sobolewski, W. Domcke, On the nature and signatures of the solvated electron in water. *Phys. Chem. Chem. Phys.* **14**, 22–34 (2012).

62. K. McNeill, S. Canonica, Triplet state dissolved organic matter in aquatic photochemistry: Reaction mechanisms, substrate scope, and photophysical properties. *Environ. Sci. Process Impacts* **18**, 1381–1399 (2016).

63. G. V. Buxton, C. L. Greenstock, W. P. Helman, A. B. Ross, Critical review of rate constants for reactions of hydrated electrons, hydrogen atoms and hydroxyl radicals (•OH/•O^-) in aqueous solution. *J. Phys. Chem. Ref. Data Monogr.* **17**, 513–886 (1988).

64. S. Bogler, K. R. Daellenbach, D. M. Bell, A. S. H. Prévôt, I. E. Haddad, N. Borduas-Dedekind, Singlet oxygen seasonality in aqueous PM_{10} is driven by biomass burning and anthropogenic secondary organic aerosol. *Environ. Sci. Technol.* **56**, 15389–15397 (2022).

65. R. Zhao, A. K. Y. Lee, L. Huang, X. Li, F. Yang, J. P. D. Abbatt, Photochemical processing of aqueous atmospheric brown carbon. *Atmos. Chem. Phys.* **15**, 6087–6100 (2015).

66. E. Melssen, D. L. Bish, Y. Losovyj, J. D. Raff, Photoreduction of nitrate to HONO and NO_x by organic matter in the presence of iron and aluminum. *ACS Earth Space Chem.* **8**, 2587–2598 (2024).

67. J. Mack, J. R. Bolton, Photochemistry of nitrite and nitrate in aqueous solution: A review. *J. Photochem. Photobiol. A Chem.* **128**, 1–13 (1999).

68. M. Rolletter, A. Hofzumahaus, A. Novelli, A. Wahner, H. Fuchs, Kinetics of the reactions of OH with CO, NO, and NO_2 and of HO_2 with NO_2 in air at 1 atm pressure, room temperature, and tropospheric water vapour concentrations. *Atmos. Chem. Phys.* **25**, 3481–3502 (2025).

69. M. Woods, F. A. F. Winiberg, C. R. Markus, K. Zuraski, R. Shannon, M. A. H. Khan, D. E. Shallcross, S. P. Sander, M. Okumura, M. McPherson, S. Smith, C. J. Percival, Pressure, temperature, and water vapor dependencies of the bimolecular rate coefficients for the reaction OH + NO + M → HONO + M. *J. Phys. Chem. A* **129**, 5579–5592 (2025).

70. J. Zhang, K. Li, T. Wang, E. Gammelsæter, R. K. Y. Cheung, M. Surdu, S. Bogler, D. Bhattu, D. S. Wang, T. Cui, L. Qi, H. Lamkaddam, I. E. Haddad, J. G. Slowik, A. S. H. Prevot, D. M. Bell, Bulk and molecular-level composition of primary organic aerosol from wood, straw, cow dung, and plastic burning. *Atmos. Chem. Phys.* **23**, 14561–14576 (2023).

71. R. Pokhrel, J. Gordon, M. Fiddler, S. Bililign, Determination of emission factors of pollutants from biomass burning of African fuels in laboratory measurements. *J. Geophys. Res. Atmos.* **126**, e2021JD034731 (2021).

72. J. Chen, C. Li, Z. Ristovski, A. Milic, Y. Gu, M. S. Islam, S. Wang, J. Hao, H. Zhang, C. He, H. Guo, H. Fu, B. Miljevic, L. Morawska, P. Thai, Y. F. Lam, G. Pereira, A. Ding, X. Huang, U. C. Dumka, A review of biomass burning: Emissions and impacts on air quality, health and climate in China. *Sci. Total Environ.* **579**, 1000–1034 (2017).

73. J. Fang, M. J. Lechowicz, Climatic limits for the present distribution of beech (*Fagus* L.) species in the world. *J. Biogeogr.* **33**, 1804–1819 (2006).

74. D. Lazic, C. Geßner, K. J. Liepe, I. Lesur-Kupin, M. Mader, C. Blanc-Jolivet, D. Gömöry, M. Liesebach, S. C. González-Martínez, M. Fladung, B. Degen, N. A. Müller, Genomic variation of European beech reveals signals of local adaptation despite high levels of phenotypic plasticity. *Nat. Commun.* **15**, 8553 (2024).

75. Y. Qin, J. Qin, X. Zhou, Y. Yang, R. Chen, J. Tan, K. Xiao, X. Wang, Effects of pH on light absorption properties of water-soluble organic compounds in particulate matter emitted from typical emission sources. *J. Hazard. Mater.* **424**, 127688 (2022).

76. M. Horbanski, D. Pöhler, J. Lampel, U. Platt, The ICAD (iterative cavity-enhanced DOAS) method. *Atmos. Meas. Tech*. **12**, 3365–3381 (2019).

77. T. S. Carter, C. L. Heald, C. D. Cappa, J. H. Kroll, T. L. Campos, H. Coe, M. I. Cotterell, N. W. Davies, D. K. Farmer, C. Fox, L. A. Garofalo, L. Hu, J. M. Langridge, E. J. T. Levin, S. M. Murphy, R. P. Pokhrel, Y. Shen, K. Szpek, J. W. Taylor, H. Wu, Investigating carbonaceous aerosol and its absorption properties from fires in the western United States (WE-CAN) and southern Africa (ORACLES and CLARIFY). *J. Geophys. Res. Atmos.* **126**, e2021JD034984 (2021).

78. A. Hecobian, Z. Liu, C. J. Hennigan, L. G. Huey, J. L. Jimenez, M. J. Cubison, S. Vay, G. S. Diskin, G. W. Sachse, A. Wisthaler, T. Mikoviny, A. J. Weinheimer, J. Liao, D. J. Knapp, P. O. Wennberg, A. Kürten, J. D. Crounse, J. St. Clair, Y. Wang, R. J. Weber, Comparison of chemical characteristics of 495 biomass burning plumes intercepted by the NASA DC-8 aircraft during the ARCTAS/CARB-2008 field campaign. *Atmos. Chem. Phys.* **11**, 13325–13337 (2011).

79. W. Song, X. Liu, Source oxygen contributions of primary nitrate emitted from biomass burning. *Sci. Total Environ.* **854**, 158736 (2023).

80. K. M. Navarro, D. Schweizer, J. R. Balmes, R. Cisneros, A review of community smoke exposure from wildfire compared to prescribed fire in the United States. *Atmosphere* **9**, 185 (2018).

81. E. J. T. Levin, G. R. M. Meeking, C. M. Carrico, L. E. Mack, S. M. Kreidenweis, C. E. Wold, H. Moosmüller, W. P. Arnott, W. M. Hao, J. L. Collett Jr., W. C. Malm, Biomass burning smoke aerosol properties measured during fire laboratory at Missoula experiments (FLAME). *J. Geophys. Res. Atmos.* **115**, D18210 (2010).

82. B. J. Sumlin, C. R. Oxford, B. Seo, R. R. Pattison, B. J. Williams, R. K. Chakrabarty, Density and homogeneous internal composition of primary brown carbon aerosol. *Environ. Sci. Technol.* **52**, 3982–3989 (2018).

83. C. Li, Z. Ma, J. Chen, X. Wang, X. Ye, L. Wang, X. Yang, H. Kan, D. J. Donaldson, A. Mellouki, Evolution of biomass burning smoke particles in the dark. *Atmos. Environ.* **120**, 244–252 (2015).

84. Y. Xu, P. Liu, S. Ma, W. Pei, S. Pang, Y. Zhang, Efflorescence kinetics of aerosols comprising internally-mixed ammonium sulfate and water-soluble organic compounds. *Atmos. Environ.* **274**, 119007 (2022).

85. M. R. Canagaratna, J. L. Jimenez, J. H. Kroll, Q. Chen, S. H. Kessler, P. Massoli, L. H. Ruiz, E. Fortner, L. R. Williams, K. R. Wilson, J. D. Surratt, N. M. Donahue, J. T. Jayne, D. R. Worsnop, Elemental ratio measurements of organic compounds using aerosol mass spectrometry: Characterization, improved calibration, and implications. *Atmos. Chem. Phys.* **15**, 253–272 (2015).

86. H. Chin, K. S. Hopstock, L. T. Fleming, S. A. Nizkorodov, H. A. Al-Abadleh, Effect of aromatic ring substituents on the ability of catechol to produce brown carbon in iron(III)-catalyzed reactions. *Environ. Sci. Atmos.* **1**, 64–78 (2021).

87. P. R. Duchowicz, J. F. Aranda, D. E. Bacelo, S. E. Fioressi, QSPR study of the Henry's law constant for heterogeneous compounds. *Chem. Eng. Res. Des.* **154**, 115–121 (2020).

88. A. C. Vandaele, C. Hermans, P. C. Simon, M. Carleer, R. Colin, S. Fally, M. F. Mérienne, A. Jenouvrier, B. Coquart, Measurements of the NO_2 absorption cross-section from 42 000 cm^{-1} to 10 000 cm^{-1} (238-1000 nm) at 220 K and 294 K. *J. Quant. Spectrosc. Radiat. Transf.* **59**, 171–184 (1998).

89. E. P. Gardner, P. D. Sperry, J. G. Calvert, Primary quantum yields of NO_2 photodissociation. *J. Geophys. Res. Atmos.* **92**, 6642–6652 (1987).

90. O. Wild, X. Zhu, M. J. Prather, Fast-J: Accurate simulation of in- and below-cloud photolysis in tropospheric chemical models. *J. Atmos. Chem.* **37**, 245–282 (2000).

91. H. Bian, M. J. Prather, Fast-J2: Accurate simulation of stratospheric photolysis in global chemical models. *J. Atmos. Chem.* **41**, 281–296 (2002).

92. S. D. Eastham, D. K. Weisenstein, S. R. H. Barrett, Development and evaluation of the unified tropospheric-stratospheric chemistry extension (UCX) for the global chemistry-transport model GEOS-Chem. *Atmos. Environ.* **89**, 52–63 (2014).

93. S. J. Pai, C. L. Heald, J. R. Pierce, S. C. Farina, E. A. Marais, J. L. Jimenez, P. Campuzano-Jost, B. A. Nault, A. M. Middlebrook, H. Coe, J. E. Shilling, R. Bahreini, J. H. Dingle, K. Vu, An evaluation of global organic aerosol schemes using airborne observations. *Atmos. Chem. Phys.* **20**, 2637–2665 (2020).

94. E. G. Schnitzler, N. G. A. Gerrebos, T. S. Carter, Y. Huang, C. L. Heald, A. K. Bertram, J. P. D. Abbatt, Rate of atmospheric brown carbon whitening governed by environmental conditions. *Proc. Natl. Acad. Sci. U.S.A.* **119**, e2205610119 (2022).

95. A. P. Sullivan, R. P. Pokhrel, Y. Shen, S. M. Murphy, D. W. Toohey, T. Campos, J. Lindaas, E. V. Fischer, J. L. Collett Jr., Examination of brown carbon absorption from wildfires in the western US during the WE-CAN study. *Atmos. Chem. Phys.* **22**, 13389–13406 (2022).

96. D. K. Farmer, S. M. Kreidenweis, WE-CAN: HR-ToF-AMS fine-mode aerosol composition data (UCAR/NCAR Earth Observing Laboratory, 2019); 10.26023/MM2Y-ZGFQ-RB0B.

97. A. P. Sullivan, WE-CAN: Particle Into Liquid Sampler 1 (PILS1) three second integrated WSOC data (UCAR/NCAR Earth Observing Laboratory, 2019); 10.26023/9H07-MD9K-430D.

98. S. R. Hall, K. Ullmann, WE-CAN: HARP (HIAPER Airborne Radiation Package) data (UCAR/NCAR Earth Observing Laboratory, 2019); 10.26023/FQKW-QG7M-Q90B.

99. Q. Peng, Secondary HONO Box Modeling Data for WE-CAN (OSF, 2022); osf.io/ncs52.
